# Lipid-driven alignment and binding of p7 dimers in early oligomer assembly

**DOI:** 10.1371/journal.pcbi.1013736

**Published:** 2025-11-25

**Authors:** Oluwatoyin Campbell, Dina Dahhan, Viviana Monje

**Affiliations:** 1 Department of Chemical and Biological Engineering, School of Engineering and Applied Sciences, University at Buffalo, Buffalo, New York, United States of America; 2 Jacobs School of Medicine and Biomedical Sciences, University at Buffalo, Buffalo, New York, United States of America; University of Maryland School of Pharmacy, UNITED STATES OF AMERICA

## Abstract

Proteins interact with lipid membranes to facilitate important cellular processes that underlie health and disease. Transmembrane proteins like ion channels are often composed of bound monomers forming specific contacts within the bilayer. However, molecular mechanisms of channel assembly are scarce. Understanding the role of lipids in this process may help further explain assembly of oligomeric proteins, which are often clinical drug targets. Using the hepatitis C virus p7 hexamer as a representative of proteins with complex transmembrane topology, this work characterizes early lipid-driven dimerization using molecular dynamics simulations. Comparing dimer interactions in aqueous solution versus on a lipid membrane model reveal that protein-lipid interactions critically guide inter-protein residue alignment and binding. Hydrophobic contacts and hydrogen bonding between key residues and phosphatidylcholine/phosphatidylinositol lipids drive essential helix interactions that promote p7 oligomerization, particularly involving the first helix. This study demonstrates that membrane lipids are essential, dynamic contributors to protein binding and aggregation in cellular membranes.

## Author summary

The dynamic interplay of proteins and lipids triggers protein conformational changes that precede their function. In the context of transmembrane protein assembly, pioneering works have proven involvement of protein chaperones in the process, but there has been little exploration of how lipids may facilitate the embedding of these transmembrane channels into cellular membranes. As an initial case study of homo-oligomeric, complex-topology channels, we examine the dimerization of the p7 protein of hepatitis C, which has been identified as a potential drug target that plays key but under-investigated roles in lipid mismanagement in liver cells. To capture interactions driving this mechanistic process in fine detail, we utilize atomistic molecular dynamics simulations as the main method of investigation. Our results show that membrane lipids play non-trivial roles in facilitating protein-protein binding during dimerization. This provides an initial step towards unraveling the full picture of how lipids are active participants in the formation of protein assemblies.

## Introduction

Protein and lipid interactions are fundamental contributors to cellular processes in health and disease. Lipid structural and compositional diversity in cell membranes provide a platform for protein interactions which underlie their function [1]. This involves changes to the structure of both proteins and lipids, which leads to the alteration of the surrounding lipid content near the protein and formation of sub-domains of different physical properties in the membrane [2–6]. These spatial and conformational changes are indicators of the influence of biochemical interactions and the cellular environment on protein structural dynamics and activity [1,7].

A class of proteins that has been well studied is transmembrane proteins, which contribute to cellular mechanisms of various types. Ion channels are a sub-class of membrane-embedded proteins that depend on voltage or ligand stimuli to move ions through lipid membranes [8–10]. Specific lipids within the immediate membrane environment can act as ligands to prompt opening and closure of the channel in different processes [11]. These structures are proposed to initiate by association of individual monomers, with folding happening alongside interactions between proteins and lipids to facilitate further aggregation during the assembly process [12]. Protein insertion and folding in the ER has been studied for single- and multi-pass membrane proteins; both cotranslational and posttranslational insertion are assisted by host factors such as Sec61, Get3, ER membrane protein complex (EMC) and other insertases [13,14]. These chaperones enable membrane proteins to overcome the energetic barriers associated with localizing hydrophobic transmembrane domains (TMDs) flanked by polar and positively charged residues within the bilayer. Further exploration of protein quaternary structure formation is also gaining more traction, with a recent study of the dimerization of *E. Coli* inner membrane protein EmrE that suggests SecYEG translocon assists in dimer formation [15]. However, oligomerization of helical proteins that form complex topologies across the transmembrane region have hardly been studied or elucidated in molecular detail.

**Fig 1:**
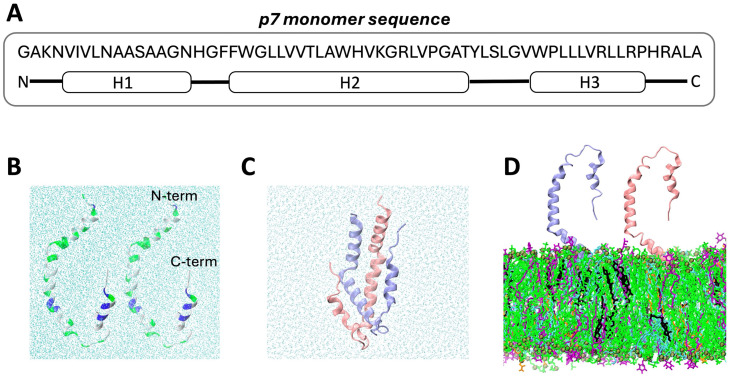
Initial simulation coordinates. A) p7 amino acids and their corresponding position within the protein. B) Two p7 monomers separated (*Sep* model) and C) extracted from the hexameric structure PDBID 2M6X (initial *Bound* model configuration). D) Two monomers initially attached by N-termini to the surface of a complex membrane (*Surface* model) containing DOPC (in green), DPPE (in blue), POPI (in purple), Cholesterol (in black) and DOPS (in orange) (55:21:11:9:4 mol%). Monomers are in the initial positioning seen in replica 1. Water atoms shown with sky blue. Proteins are shown with cartoon representation and differentiated in pink and ice-blue (p1 and p2, respectively); when shown, non-polar residues in white, polar in green and cationic in blue. In snapshots, water and ions are omitted for clarity.

In the context of disease, viroporins are ion channels that enable virus production by contributing to various steps in the viral life cycle [16–18]. In hepatitis C, the p7 viral ion channel is made up of six individual helical proteins containing 63 amino acids each (Fig 1A). The channel structure stands out because the six monomers intricately intertwine, such that helix 1(H1) and helix 2 (H2) run through the ER to form the pore, and H3 is found on the exterior, where it packs against H2 of the i + 2 and H1 of the i + 3 monomer [19]. It is possible that such interconnected structures with complex topology may be achieved by exogenous or host chaperones, given the evidence that p7 engages in interactions with other HCV genome proteins such as NS2 and E2 to facilitate virus replication and assembly, in addition to the host interferon protein IFI6–16 that promotes immune suppression [20]. Nonetheless, the role of lipids in mediating protein-lipid and protein-protein interactions may be larger than initially assumed. Our previous study on the interaction mechanisms of p7 with model bilayers shows deeper insertion of a p7 monomer in the presence of phosphatidylserine (PS) lipids, implying the ability of lipids to act as molecular chaperones that drive membrane entry via hydrophobic and electrostatic interactions [21]. Similarly, an *in vitro* study shows spontaneous insertion of the potassium channel-forming protein KcsA into liposome membrane models mediated by interactions with positively charged residues [22]. Biophysical experiments and molecular simulations also showed membrane surface absorption of the intertwined, trimeric HR1 domain of the caveolin1 protein, illustrating that membrane surface charge and cationic protein residues induce favorable membrane localization of oligomers [23].

p7 has been shown to play important roles in facilitating continuous virus production. Its influence on viral assembly, lipid droplet metabolism and localization near the lipid bodies have been well established, with these functions enabled through collaborations with other hepatitis C virus (HCV) proteins [24–26]. Experimental investigations have revealed the role of anionic lipids in sustaining channel structure and membrane permeabilization activity [27,28]. The mechanism of p7 has been suggested to rely on the interplay between lipids and proteins that alter structural and mechanical properties of the local lipid environment. Molecular studies on p7 have focused on the role of specific protein residues in sustaining the spatial conformation of p7 monomers and hexameric channels within the membrane. Arg33 and Arg35 interact with negatively-charged lipid headgroups to sustain the transmembrane structure of p7 [28], cholesterol prompts a cascade of interactions that position His17 properly within the channel pore [6] and Tyr42 and Tyr45 sustain the kinks in the monomer membrane-embedded structure by increasing charged lipids presence nearby [29]. However, despite these key contributions, the assembly process of the p7 channel remains yet unclear. Improved understanding can aid in the design and development of therapeutics that target the action of homo-oligomeric membrane channels during viral disease pathogenesis.

Given the evidence of transmembrane assembly of amphiphilic proteins without the assistance of other biomolecules but the membrane itself, we hypothesize that p7 assembly can be mediated by membrane lipids. A promising tool to probe this research question is molecular dynamics (MD) simulations. This technique utilizes statistical mechanics calculations of interaction forces to predict behavior of molecules in their native environments, and has proven to be advantageous for modeling and highlighting protein-protein and protein-lipid interactions and mechanisms at the microscale [30–32]. Herein, we focus on elucidating the posttranslational dimerization mechanism of p7 dimers prior to membrane entry by running microsecond-long classical MD simulations of two p7 monomers in water and near a membrane model for the endoplasmic reticulum (ER). Results consistently show that dimerization patterns of p7 heavily rely on hydrophobic and polar interactions with zwitterionic phosphatidylcholine (PC) and anionic phosphatidylinositol (PI) membrane lipids, specifically for protein membrane adsorption. These interactions provide a strong foundation that fixes p7 monomers in place to facilitate important hydrophobic interactions between them, especially in the first helix of its N-terminus. This work gives a detailed account of the function of apolar and charged lipids in mediating oligomeric protein structures.

## Materials and methods

To probe the effect of lipid composition on dimerization of p7 monomers, three different models were simulated: (i) the monomers initially separated in water (*Sep* model), (ii) a control system containing two monomers in water in the experimentally determined channel conformation from PDBID 2M6X (*Bound* model), and (iii) the monomers initially separated on the surface of an anionic-charged membrane (*Surface* model). Fig 1B-1D illustrates the setup of each model, while S1 Fig shows the different starting positions of the monomers in the *Surface* model. The *Bound* model serves as control, and a representation of the relative conformation of a p7 dimer in their final channel configuration. The *Sep* and *Surface* systems are compared against the *Bound* configuration to elucidate the effect of lipids on protein-protein dynamics at initial stages of assembly. The membrane present in the *Surface* systems is based on the ER [27,33], containing 600 lipids per leaflet with 55% dioleoyl-phosphatidylcholine (DOPC), 21% dipalmitoyl-phosphatidylethanolamine (DPPE), 11% 1-palmitoyl-2-oleoyl-phosphatidylinositol (POPI), 9% cholesterol and 4% dioleoyl-phosphatidylserine (DOPS) lipid molecules. S1 Table outlines relevant details of all simulated systems, including the respective number of replicas and simulation length. A total of 6.4 μs of trajectories using classical all-atom MD simulations were collected for this study.

The *Bound* and *Sep* models were constructed by placing two monomers from the 2M6X PDB channel structure in an aqueous solution box using CHARMM-GUI *Solution Builder* [34–36]. After the 4-step relaxation protocol generated by the builder, each model was run for 400 ns. For the *Surface* model, the bilayers were set up using the CHARMM-GUI *Membrane Builder* [34–38]. After completing the 6-step relaxation protocol generated by CHARMM-GUI, each membrane was equilibrated for 200 ns. Afterwards, an in-house bash script was used to merge the equilibrated membrane and monomers coordinates and run for 1 ms each. All systems were fully hydrated and neutralized with 0.150 molar KCl. The *Bound* model was placed in aqueous environment instead of inside a lipid membrane to characterize the natural interaction of dimers when the bilayer is not present. Given the lack of preceding studies on how and where the p7 dimers and channel assemble, this study was designed to probe the role of lipids on protein interactions and early assembly dynamics. As the simulation progresses, the p7 dimers in the *Bound* conformation deviate from the coordinates in the crystal structure of the channel in a lipid environment (S2 Fig), yet it remains a fit reference state for the *Sep* and *Surface* models that start in solution.

The CHARMM36m forcefield [39,40] which has been extensively validated with resulting structure of protein and lipids from experiments was applied in all simulations. The TIP3 model [41] was used to represent water molecules in the systems. The GROMACS package [42] was used to perform molecular dynamics on each model system. A timestep of 2 fs was chosen, which allows appropriate capture of vibrations of covalent bonded hydrogens constrained by the LINCS algorithm [43]. Electrostatic and van der Waals (vdW) non-bonded interactions were calculated with the Particle Mesh Edwards [44] and Verlet [45] algorithms. vdW interactions were modeled based on a Lennard-Jones potential force-switch function with a soft cutoff starting from 1.0 nm. Integration was carried out with the leap-frog integrator [46]. Temperature and pressure were set to 315.15K and 1 bar to capture body temperature and atmospheric pressure conditions. The Nose-Hoover [47,48] and Parrinello-Rahman [49,50] thermostat and barostat were used, with coupling times of 1.0 and 5.0 ps respectively. Compressibility was set at 4.5e-5 bar^-1^, based on that of water. Groups containing protein, membrane and water atoms were coupled separately to the 315 K temperature bath, and pressure was controlled semi-isotropically.

Analysis of resulting data was performed with in-house scripts that feature built-in tools from GROMACS and VMD, and Python libraries such as Numpy, Pandas, and MDAnalysis [51,52]. Analysis was computed using block averaging with respective standard errors for the latter half of the trajectories, utilizing at least 200ns of trajectory. A distance cutoff of 14 Å was used to define contacts between atoms to ensure sufficient sampling of lipids and/or amino acids. This is an appropriate range in MD simulations that captures interactions between protein carbons and lipid phosphorus atoms. A full description of key analysis can be found in the supporting information (S1 Text), and sample scripts are publicly available at https://github.com/monjegroup/p7-dimer. In interaction analyses, interhelical interactions are referred to in the sequence of the first, then second monomer, e.g., H3-H2 refers to helix 3 of monomer 1 interacting with helix 2 of monomer 2. Snapshots were rendered with VMD [53]; in all instances, water and ions are not shown for clarity.

## Results

### Dimer conformational changes are mediated by membrane lipids

**Fig 2:**
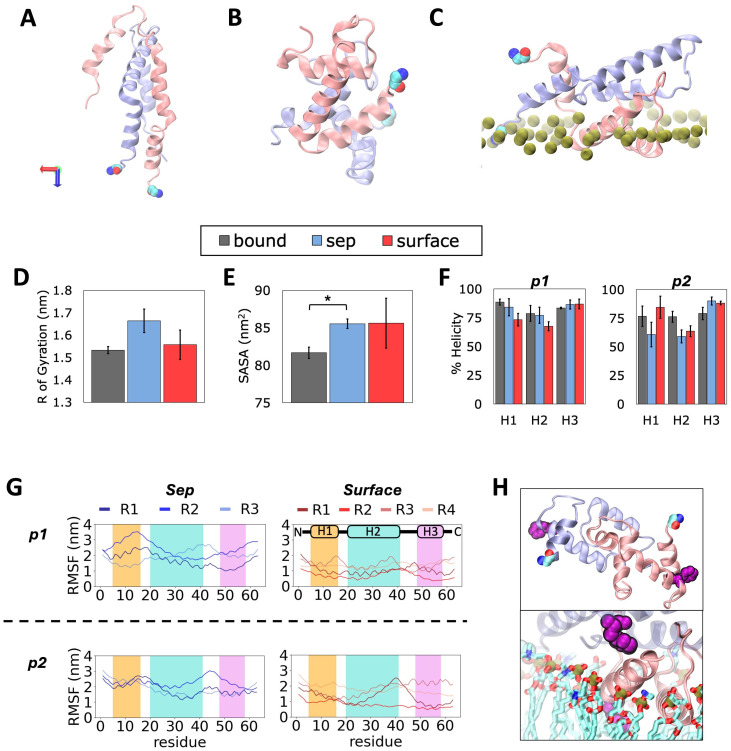
Protein conformational dynamics induced by presence of a lipid membrane. **A)** Initial conformation of dimers in the *Bound* reference model obtained from the p7 channel structure (available as PDB 2M6X). **B)** Representative final conformations of dimer structures formed in water (*Sep* model) and **C)** lipid (*Surface* model) environments. **D)** Average R_g_, **E)** SASA and **F)** protein % helicity in each model. **G)** RMSF of protein residues in *Sep* and *Surface* replicas. **H)** Snapshot of the Phe20, shown in purple, in helix 2 (H2), illustrating difference in localization in *Sep* and *Surface* models. Monomer 1 (p1) shown in pink, monomer 2 (p2) in ice-blue and N-terminus end indicated with van der Waal representation. Phosphorus atoms are shown in green and lipid tails in cyan. In all snapshots, water and ions hidden for clarity. Error bars represent standard error across replicas, and “*” indicates significant difference in means (p < 0.05).

After simulations were completed, the structural and conformational changes within the monomers were first evaluated. The *Sep* and *Surface* model systems (Fig 1) equilibrated within the first half of the respective trajectory, as seen from the root mean square deviation (RMSD) time series of monomer 1 and monomer 2 (referred to as p1 and p2 in S3A and S3B Fig), which also exhibit converged RMSF profiles for most replicas (S4 Fig). Therefore, the last 200ns of the *Sep* models, and the last 500 ns of the *Surface* models were used to calculate averages and standard errors of all properties reported for each replica. The RMSD of both p7 monomers is greater in the *Sep* model compared to the *Surface* model (p1: 1.40 ± 0.03 nm versus 1.29 ± 0.03 nm; p2: 1.37 ± 0.04 nm versus 1.22 ± 0.03 nm), corresponding to more notable change in secondary structure for the proteins in water. To compare both structures to the monomer conformation in the channel, the RMSD was also calculated using the *Bound* coordinates as the reference (S3C Fig). The *Bound* model acts as a reference, with a conformation that initiates as the coordinates extracted from PDBID 2M6X (Fig 2A). The difference is lower for monomer 2 in the *Surface* model, indicating more similarity to the *Bound* configuration in the presence of lipids.

The representative final conformations in each model can be seen in Fig 2; p1 and p2 monomers are colored in pink and ice-blue, respectively. Compared to the *Bound* model (Fig 2A), the *Sep* model exhibits a globular dimeric structure (Fig 2B), while the monomers stretch out as they interact with the membrane interface in the *Surface* model (Fig 2C). To determine the depth of entry of each protein residue, the z-component of the distance between its center of mass (COM) and the average position of the lipid phosphorus atoms was calculated over the last 500 ns of trajectory. In general, the monomers remained above the membrane hydrophobic phase. In p1, residues 46–54 (Gly-Val-Trp-Pro-Leu-Leu-Leu-Val-Arg) are found closest to the membrane lipids, while p2 has Gly1 and Ala63 (N- and C-termini respectively) closest to the membrane (S5 Fig).

When compared with the *Bound* model, the Radius of gyration (R_g_) of the *Surface* model appears lower, implying a more compact dimer structure, while that of the *Sep* model is larger (Fig 2D). However, the difference between *Sep* and *Bound* is not statistically significant (p > 0.05). It is worth noting that the *Sep* model has a statistically greater solvent accessible surface area (SASA) than the *Bound*, while the *Surface* model does not (Fig 2E). This implies that the exposure of residues to water is greater in the *Sep* model, and may act as a deterrent against proper alignment of monomers. To probe how much of the secondary structure is retained in the monomers after inter-protein contacts, the helical percentage of each of the three helices in each monomer was measured in the last half of the trajectories and reported in Fig 2F. These helices, referred to as H1, H2 and H3 hereafter, correspond to residues Val5 to Asn16, Phe20 to Thr41 and Trp48 to Pro58 in the p7 structure [19] (see. Fig 1A). The helical structure of p1 in the *Sep* model is comparable to the *Bound* reference, while that of *Surface* p1 loses a small amount of helicity in H1 and H2. In contrast, when compared with the *Bound* structure, *Sep* p2 loses while *Surface* p2 gains helicity in H2 and H3, respectively. The average helical content of H1 of the *Surface* model is greater than both *Bound* and *Sep* models.

To learn more about the dynamics of individual residues within the dimers, the root mean squared fluctuation (RMSF) of p7 residues was analyzed over the entire trajectory of each model replica and reported in Fig 2G. In general, the RMSF is greater for *Sep* replicas than the *Surface* replicas. Replicas do not exhibit the exact same RMSF profile, which is expected; yet, similar trends of peaks and minima can be identified for each model, particularly for residues found at the end of H1, which show higher fluctuation in *Sep* model versus the *Surface* model. *Sep* H2 helix has decreasing RMSF as opposed to *Surface* H2 that shows increasing RMSF along the helix, while H3 displays no sustained trend, implying its lower involvement during dimer formation in water or on the membrane surface. This is expected as H3 are the farthest apart between the monomers in the reference structure (Fig 2A). The peaks and minima in the *Surface* model correspond to residues that interact with water or membrane lipids, respectively. Take for example, Phe20 at the start of H2 in each monomer (Fig 2H), it faces the water phase in the *Sep* model (top panel) but it points to the membrane interior and interacts with the other monomer in the *Surface* model (bottom panel). These results show the role of membrane lipids in the formation of protein-protein contacts and conformational changes, which facilitate dimer formation.

### Lipids contribute to the residue alignment of p7 dimers

**Fig 3:**
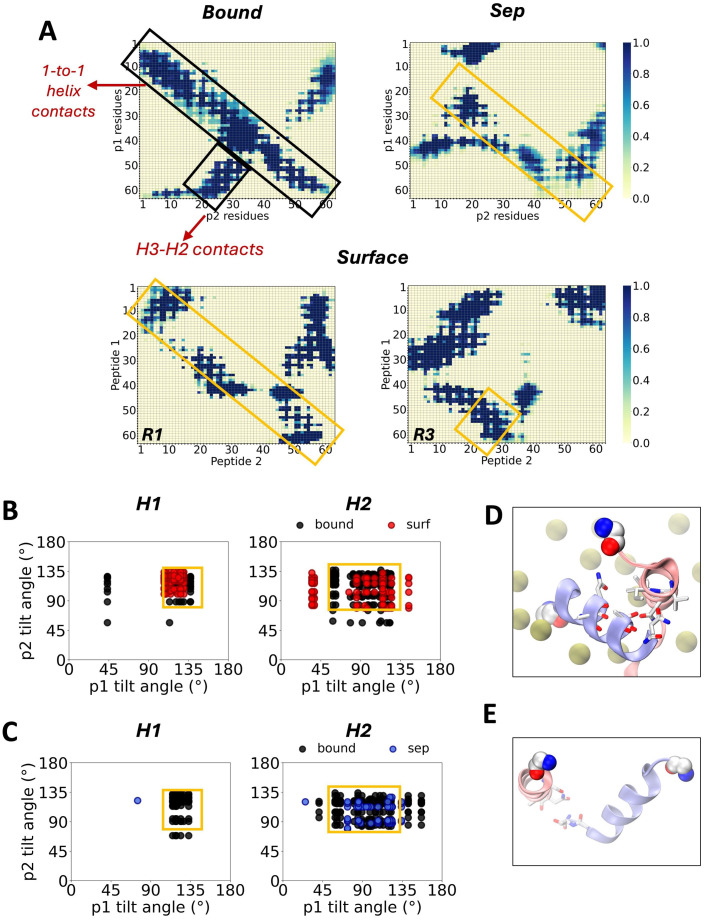
Residue contact comparison with reference dimer structure. **A)** Contact maps of residues, based on a cutoff of 14 Å. *Surface* replicas 1 (R1) and 3 (R3) shown in bottom panel. **B)** Tilt angle conformational landscapes of 1-to-1 contacting helices in p1 and p2 in representative *Surface* and **C)** *Sep* replicas. **D)** Snapshots showing helix 1 contacts in *Surface* and **E)** *Sep* representative replicas. Contacts and tilt angles indicate trends during second half of system trajectories. Target contacts conserved across all *Bound* reference model replicas (1-to-1 inter-helical contacts and helix 3-helix 2 contacts) indicated with black rectangles. Contact regions formed in *Sep* and *Surface* models indicated with yellow rectangles. Carbon, oxygen and nitrogen atoms shown in white, red and blue respectively.

To shed more light on the inter-protein alignment behavior of p7 in the studied models, an inter-residue contact analysis was carried out with a cutoff of 14 Å. The fraction of time protein contacts occurred during the equilibrated trajectories in the *Sep* and *Surface* models was compared against those of the initial 50 ns of the *Bound* reference structure. Contact maps for the full set of replicas of each model is shown in S6 Fig. The conserved contacts between monomers across all replicas of the *Bound* model is indicated with black rectangles. Fig 3A highlights main trends across models. In the *Sep* model, conserved target interactions observed in the *Bound* reference which involve 1-to-1 (i.e., H1-H1, H2-H2 and H3-H3 contacts) and H3-H2 helix contacts are reduced, with those in the H1-H1 region completely missing. In comparison, the *Surface* model containing the anionic membrane lipids exhibits contacts in the two critical regions that correspond to the reference state. Therefore, though full dimerization was not seen, the lipid membrane clearly facilitates proper H1-H1 and H3-H2 interactions.

Next, the conformational landscape of the interacting residues involved in the 1-to-1 helix alignments were further evaluated. For residues with a contact occupancy fraction of at least 0.9, the tilt angle of the side chain was measured with respect to the vector defined between alpha carbons of residues with low flexibility as determined from the trajectory movie (see S2 Table). These reference alpha carbons were as follows: Val7 and Ala14 for H1, Gly22 and Lys33 for H2 and Pro49 and Leu56 for H3. The full set of results for *Sep* and *Surface* replicas can be seen in S7 and S8 Figs, respectively. There is a conserved region across all replicas of the *Bound* model in the H1 and H2 conformation maps (indicated in yellow). The same region is observed in the corresponding maps for H2 in the *Sep* and *Surface* models, while that of H1 is only reproduced in the *Surface* model (Fig 3B and 3C). *Surface* representative snapshots show contacts facilitated by both polar and hydrophobic residues in H1 as the N-terminus of p2 anchors both helices to the membrane (Fig 3D). In contrast, H1 helices in the dimer in water are consistently apart from one another (Fig 3E), suggesting membrane lipids promote H1-H1 interactions.

When comparing contact and conformational maps of all *Surface* replicas, a unique behavior can be seen in replica 4, which has an absence of points in the conserved regions of both the contact and 2D tilt angle maps (S6 and S8 Figs). SASA and R_g_ measurements of this replica in S9A and S9B Fig show it deviates from the behavior observed in the other *Surface* replicas in that the monomers are further apart (see also S9C Fig vs Fig 2C). This may result due to the orientation of the N-terminus of p2 that points outwards to the aqueous phase, as p1 moves towards the leaflet and fully interacts with membrane lipids during initial steps of the simulation (S9D Fig); this behavior is absent in other replicas.

### Protein binding interactions are more favorable in the presence of lipids

**Fig 4:**
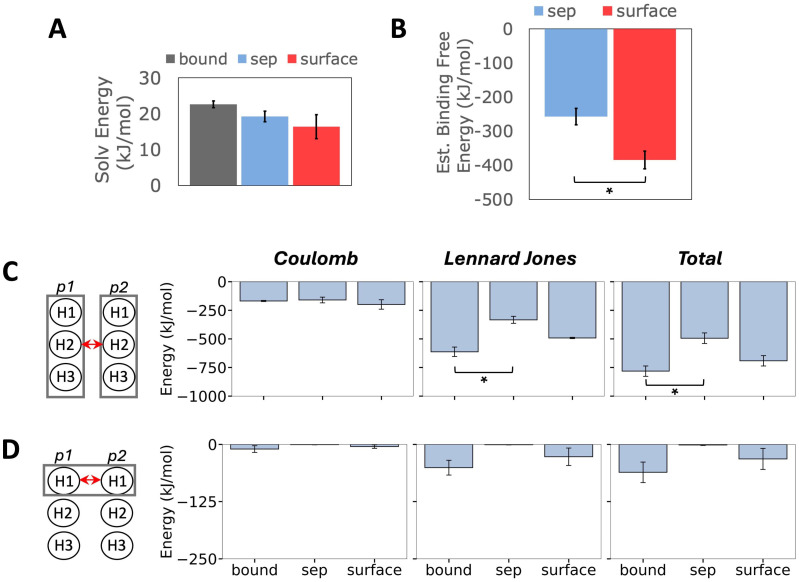
Energetics of protein interactions. **A)** Solvation energy of the formed dimer in each model. **B)** Estimate of the protein binding free energy per model, computed with the Molecular Mechanics with Generalized Born and Surface Area (MM-GBSA) approach. **C)** Coulomb (electrostatic), Lennard-Jones (hydrophobic) and total interaction energy of monomer 1 and monomer 2 and **D)** helix 1 and helix 1 in each model. These energies are shown in the first, second and third column respectively. Error bars represent standard error across replicas, and “*” indicates significant difference in means (p < 0.05). Statistics computed exclude replica 4.

The structural and conformational analyses above show that the monomers align in a manner more similar to the *Bound* reference structure in the presence of lipids. To identify key interactions that drive such a distinct dimerization mechanism in presence of lipids, the energetics of solvation, non-bonded interaction, and free energy of inter-monomer binding are reported in Fig 4. The average solvation energy is lower for the *Surface* model compared to the *Sep* model (Fig 4A), implying the cost of monomer dissociation is lower and more favorable in the presence of lipids. The reference structure from the channel exhibited the highest solvation energy, showing that the energy that must be overcome to detach the binding monomers in the *Bound* model is greater than *Sep* and *Surface* models.

The binding free energy was estimated using the Molecular Mechanics Generalized Born Surface Area (MM-GBSA) method, further described in S1 Text. These estimates take into account the solvation energy described by the Generalized-Born equation and the interaction energy of in-vacuum binding; using the gmx_MMPBSA software [54], the free energy difference with respect to the bound state was determined. The protein binding free energy can be broken down into enthalpy and entropy terms (∆H and -T∆S respectively). Measurements here account only for enthalpic contributions due to the large standard deviation in the predicted entropy. Note that -T∆S is expected to remain stable given the reduced conformational freedom of the monomers after binding in the *Sep* and *Surface* models. Fig 4B shows that the binding free energy is statistically more favorable in the *Surface* model compared to the *Sep* model, supporting the hypothesis that membrane lipids are important drivers for inter-protein binding and formation of p7 dimers.

To evaluate the contribution from non-bonded interactions, the electrostatic (Coulomb) and hydrophobic (Lennard Jones) energies were determined using the *gmx rerun* command in GROMACS. There is little difference in electrostatic contributions across models. In contrast, hydrophobic interactions are markedly larger in the *Surface* model, leading to statistically more favorable interactions between the monomers at the membrane surface (Fig 4C). The total H1-H1 non-bonded interaction energy in the *Surface* model is comparable to the *Bound* reference, while that of *Sep* model is effectively zero (Fig 4D). This shows more favorable binding between monomers at the membrane leaflet, which led to more accurate structure and conformation of the *Surface* dimer structure. This suggests that hydrophobic interactions play an important role in driving protein alignment for the formation of p7 dimers.

Non-zero estimates of the non-bonded energy for other helical interactions are also shown in S10 Fig. As in the full monomer case, the greatest differences between the *Sep* and *Surface* models arise to favor hydrophobic interactions. Of note, H2-H2 interactions are less favorable in both *Sep* and *Surface* models than in the reference structure, while H3-H3 interactions are overrepresented in both compared to the reference structure. Lastly, H2-H3 interactions are miniscule, while H3-H2 interaction favorability falls short in both *Sep* and *Surface* models in comparison to the *Bound* structure. This difference in H2-H3 vs H3-H2 interactions is understandable, as though the sequences of the two monomers are identical, each helix and residue sees a different chemical environment based on individual distance from other molecules in the system.

### Neutral and anionic lipids enhance dimer alignment and anticorrelated protein-lipid motions

**Fig 5:**
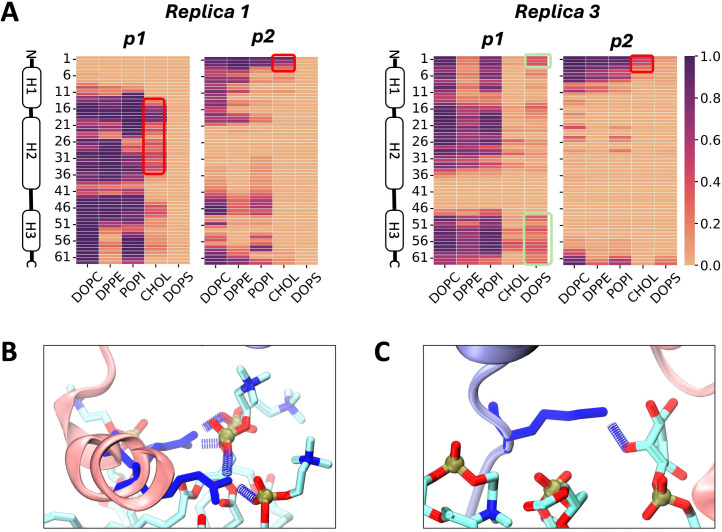
Protein-lipid interactions in the *Surface* model. **A)** Frequency of contacts between each protein residue and lipid species in the second half of the trajectory, based on a cutoff of 14 Å. Results shown are representative *Surface* replicas which had the best alignment of the dimers. Cholesterol and DOPS contacts highlighted with red and light green rectangles respectively. Snapshots illustrating some important hydrogen bonds: **B)** Arg residues in helix 3 of p1 with DOPC lipids, and **C)** Lys residue in N-terminus of p2 with a POPI lipid. Arg and Lys residues shown in dark blue licorice representation. Lipid carbon, oxygen, nitrogen and phosphorus atoms are shown in cyan, red, blue and gold respectively.

To determine specific protein-lipid interactions during dimer assembly, the contact frequency of these structures was analyzed during the last 500 ns of trajectory, using 14 Å as the cutoff distance. The results for the *Surface* replicas with the most similar inter-protein contacts compared to the *Bound* structure are shown in Fig 5A, replica 1 showcases 1-to-1 helix alignment, and replica 3 that of H3-H2 alignment. Both replicas feature notable presence of DOPC, POPI and DPPE contacts with p1, the closest monomer to the membrane. Cholesterol (indicated in red) interacts mostly with H1 and H2 residues in p1 and the N-terminus of p2, all of which enter deeper into the membrane interior compared to others (S11 Fig), and stabilize the protein dimerization process. DOPS contacts show up only in the case of H3-H2 alignment as it interacts with residues at the N-terminus and H3 of p1 (indicated in green). Our results suggest this interaction may be needed to stabilize H3-H2 alignment for accurate dimer formation.

The hydrogen bonding frequency between protein residues and lipids was also calculated for the last 500ns of trajectory in the *Surface* model, based on a distance and angle cutoff of 3.2 nm and 30^o^, respectively. The representative replicas show hydrogen bonds involving arginine residues in H3 and lysine at the tip of H1 with DOPC and POPI lipids (Fig 5B and 5C). Once again, DOPS notably enhances H3-H2 alignment shown in replica 3, with p1 forming well-sustained hydrogen bonds with Lys3, Arg57 and Arg60 (S12 Fig). In replica 1, additional H-bonds formed with the 15–21 (Gly-Asn-His-Gly-Phe-Phe-Trp) sequence in the first loop after H1, and Trp30, His31 and Arg35 in H2 may be aiding the formation of proper H1-H1, H2-H2 and H3-H3 contacts (S12 Fig). The N-termini H-bonds strengthen monomer-lipid interactions to sustain H1-H1 alignment between the monomers.

Dynamic cross correlations (DCC) analysis was also conducted to investigate correlated behavior in protein-lipid dynamics using an adaptation of the algorithm reported in Tekpinar et al. [55]. This involved tracking the displacement of interacting protein alpha carbons and lipid phosphate atoms, and after which the time average of the dot product of individual carbon-phosphate combinations is computed. To minimize empty selections during the calculation, protein-lipid contacts were selected based on a cutoff of 20 Å. Positive correlations indicate unidirectional movement of protein-lipid pairs, while negative correlations indicate movement in opposite directions.

**Fig 6:**
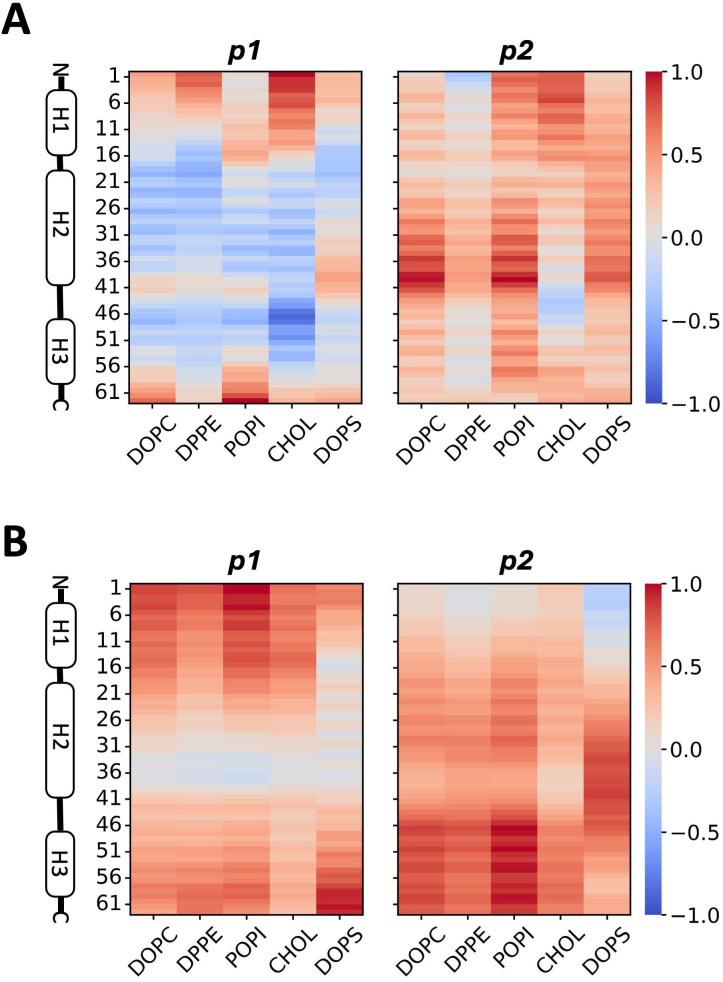
Correlation of protein and lipid motions in the *Surface* model. **A)** Map of dynamic cross correlations (DCC) of each monomer with each contacting lipid species in the replica 1 and **B)** replica 3 during the entire simulation trajectory. Negative correlation is represented in blue, and positive correlation in red. Contact is defined within a cutoff of 20 Å.

Fig 6 shows key results; the two *Surface* replicas with the greatest similarity to the *Bound* reference model stand out by displaying anticorrelated motions. When 1-to-1 helix alignment is present, the motions of residues 11–56 in p1 are anticorrelated with all 5 lipids, motions of residues 1–4 and 46–50 in p2 are anticorrelated with DPPE, and motions of residues 41–47 in p2 are anticorrelated with cholesterol (Fig 6A). On the other hand, when H3-H2 contacts are present, motions of residues 32–39 in p1 are anticorrelated with the top 4 lipids, motions of residues 14–39 in p1 are anticorrelated with DOPS, and motions of residues 1–16 in p2 are anticorrelated with DPPE and DOPS (Fig 6B). This is in contrast with the fully positive correlation between all residue-lipid combinations observed for the replicas that did not show target inter-protein binding (S13 Fig). These results imply that anticorrelated protein-lipid movement may be a marker for protein dimer assembly.

## Discussion

Protein assembly mechanisms of transmembrane channels remain underexplained for the most part. This stems from existing challenges in examining the dynamic changes of membrane protein structures at high resolution. Vesicles and lipid detergents used in experimental investigations are often limited to simple mixtures of membrane lipids compared to more complex models of the diverse lipidome of cells [56]. Techniques such as NMR, Xray crystallography and cryo-electron microscopy cannot capture detailed dynamics seen below the microsecond timescale, and cannot capture the specificity of lipid influence on these processes [57,58]. In structural studies of ion-channel forming proteins, efforts have focused on exploring pore characteristics within the membrane core [59–62], or the monomers already set in oligomeric form prior to interacting with the membrane interface [63]. Given the lack of understanding on how these structures oligomerize, we set out to explore the molecular mechanism by which a model ion channel with complex topology forms. Specifically, we investigated the mediatory role of membrane lipids in facilitating protein-protein interactions in the formation of p7 dimers from HCV. This approach is based on preceding experimental and computational studies that firmly establish membrane composition and their interaction with specific protein residues as determining factors for proper protein insertion and oligomerization within membranes [21–23]. Here, we examine two identical monomers following the premise that ion channel assembly mechanism starts with the binding of two monomers into a dimer [12]. Our study analyzes long, unbiased MD simulation trajectories of two p7 monomers in aqueous solution and at the membrane interface of a complex membrane model for the ER.

Evaluation of structural and conformational properties of the monomers emphasized differences in dimerization of the *Sep* model, which contains the monomers in water, and the *Surface* model, where the p7 monomers are near a model bilayer. Helicity of p7, particularly for H1 and H2, is modulated in different ways, depending on the orientation of the aligned structure and its chemical environment. Membrane lipids facilitate more compact dimer conformation, with increased helicity for H1 in one of the monomers versus its water counterpart. Monomers of amphiphilic proteins retain more helical structure at the membrane interface than in solution [21,63], and exhibit a reduced tendency to unfold when in oligomeric form [63]. The orientation of individual residue side chains shared increased similarity to the reference *Bound* state conformations for the dimer formed near the bilayer. H1, the first helix of p7, is amphipathic, featuring both polar and non-polar protein residues. Amphipathic proteins are well-characterized as effective pore inducers within bacterial membranes in the context of antimicrobial activity [64]. The lower helicity in the amphipathic H1 of p7 observed in water is supported by studies of the aggregation mechanisms of membrane disrupting peptides from bacteria and parainfluenza, which report a negative impact of water on helical propensity [59,65]. Hence, folding dynamics of helical proteins is nuanced and dependent on their local environment.

The membrane acts as an anchor for the monomer in closer contact to the lipid headgroups, reducing residue fluctuation. The RMSF profile of the monomers showed a non-uniform adsorption behavior in both *Sep* and *Surface* models, as the residues with lowest and highest fluctuations were different in each model. Yet, differences were particularly distinct for residues at the end of H1 (orange region) in the presence of lipids, which had lower RMSF values. Inter-protein H1 contacts did not readily form in water, suggesting anchoring of at least one monomer by membrane lipids facilitates inter-helix alignment. In the fully formed hexameric channel, the first helix of p7 is found within the interior pore structure [19]. Given this eventual positioning, the amphipathic nature of this helix likely prompts dimer interactions to minimize hydrophobic strain as the protein inserts into the membrane interior.

The surrounding environment also influences protein conformations and interactions. This is demonstrated by changes in the 1-to-1 helix contacts, which led to different tilt angle combinations for individual residue side chains involved in the contact. The range of angles accessible by the residues in H1 of the nearest monomer to the membrane is more restricted than those of the other one; spanning 100^0^-145^0^ for H1 of p1, compared to 70^0^-135^0^ for H1 of p2. None of the *Sep* replicas access these conformations for correct H1-H1 alignment, while 3 out of 4 *Surface* replicas did. This implies that proper H1-H1 alignment of residue side chains between p7 monomers requires the assistance of lipid interactions. Nonetheless, the protein-protein residue contact maps of the *Surface* model lack a few clusters of contacts in key regions, indicating that alignment of p7 monomers was only partially achieved. A full alignment will require further investigation via protein structure resolution and assembly studies.

Binding free energy quantifies spontaneity of a molecular process. Here, measures of the binding free energy and its individual components such as solvation, hydrophobic, and electrostatic contributions show contact with the lipid membrane interface reducing the energetic cost of monomer binding. The contributions from electrostatic interactions were nearly equal in all model cases, while clear differences were found in hydrophobic contributions, where Lennard Jones energy was more favorable in the presence of membrane lipids. These results agree with conclusions of an experimental and computational study of the pore-forming channel Vp4 of hepatitis A virus, where mutations that reduced protein hydrophobicity led to attenuation of membrane entry and virus production [63], a coarse-grained simulation study that concluded LJ interactions contribute the most to protein dimerization compared to electrostatics for the BAR-domain protein LSP1, and this process was more favorable at the membrane interface [66], and how dimer formation of helix-containing proteins benefits from hydrophobic interactions provided by apolar bulky residues such as Leu, Ile and Trp [65,67]. Interestingly, H1-H1 interactions are favored in the presence of the membrane but H2-H2 and H3-H2 interactions were not observed in either aqueous or lipid environments, possibly because there are greater energetic barriers to be overcome to form target inter-helical contacts as the protein aggregates into higher order oligomers. The *Surface* model also did not induce deep entry of the dimeric p7 into the hydrophobic phase of the membrane compared to our previous monomer study. A replica exchange simulation study of a similar protein supports that the process of dimerization is more energetically costly than remaining in monomeric form [65], and this may explain this limited sampling of dimer insertion. The process of oligomer formation is reported to be on the order of minutes for many viral proteins as well [68]. Therefore, to fully probe the oligomerization mechanism of p7, enhanced sampling approaches will be needed.

Lipid dynamics have been shown to mediate formation of protein oligomers via lateral lipid reorganization that catalyze formation of membrane nano-domains, which lower energetic costs for protein association [69]. In this study, systems that exhibit better dimer alignment show opposite movements in the dimer alpha carbons and lipid phosphorus atoms in the vicinity of the protein. Anticorrelated dynamics are in contrast with positive correlations often shown by stable and allosteric proteins complexed with other proteins or in contact with ligands [70–72]. The results therefore suggest increased flexibility of p7 which may facilitate its progression towards the oligomeric structure; more studies are needed to explore this hypothesis.

We also probed contact and hydrogen bonding patterns between p7 and neighboring lipid species to elucidate what type of interactions assist inter-protein binding at the membrane surface. The greater contact area of the monomer closer to the membrane and sustained contact of terminal residues of the monomer farther from the membrane seem to be key for proper peptide formation, with DOPC, POPI and DOPS playing important roles. In general, zwitterionic PC and anionic PI lipids contribute the most to p7 interactions, including sustained hydrogen bonds with cationic Arg and Lys residues. PS contributions are centered around pinning down H3 to the membrane surface. This is expected and in line with p7 structure, given that H3 contains the greatest presence of cationic Arg residues. The role of PS in facilitating protein-protein interactions during viral protein oligomerization is also reported in an experimental study of the VP40 matrix protein of the Marburg virus, where PS was highlighted as important for virus assembly and maturation [67]. Zwitterionic lipid concentration has also been noted to modulate organization of TMDs in vitro, with PE cited as a direct lipochaperone for the E. Coli LacY transporter without the need of other cellular factors [73,74], implying its ability to drive assembly of protein quaternary structures without assistance.

Notably, our study also showed sustained cholesterol contacts with the p7 terminal ends and majority of H2 residues when there was 1-to-1 helix alignment. This implies a role of cholesterol in ensuring stable protein conformation that may drive oligomer formation, in agreement with results from a fluorescence spectroscopy study that shows cholesterol drives membrane penetration and oligomer formation of the amphiphilic viral fusion peptide of SARS-CoV [75]. Interestingly, this fluorescence study also shows low and high cholesterol concentration modulate the two roles respectively. Hence, it is possible that local membrane transitions between cholesterol enriched and depleted regions are relevant for protein oligomerization.

Previous experimental studies of viral proteins show that higher concentration of monomers drives faster oligomer formation as they pool together [76], with greater efficiency observed when the subunits originate from the same translated polyprotein product [77]. This suggests that close proximity between monomers is the catalyst for efficient dimerization, which informed the setup utilized in this work. Whether these monomers aggregate at the membrane surface before or after transmembrane localization remains to be determined, though an insertion-dimerization mechanism seems plausible for complex viral protein channels like p7. Such assembly pathway would require transmembrane diffusion of protein subunits prior to oligomerization. This may be challenging, given the protein-saturated nature of the ER which stems from its roles in protein synthesis and quality control [78,79]. Characterization of this assembly pathway may be worthwhile to establish comprehensive understanding of the molecular mechanisms that drive transmembrane protein oligomer assembly.

## Conclusion

The process of oligomeric assembly of ion-channel forming proteins is not fully understood, and there are currently little to no investigations that outline the detailed mechanism of this important biological phenomenon. In an effort to clarify this, this work probes the unique role of lipids in the initial step of oligomerization of the HCV p7 viroporin as a case study that is characterized by an intertwined transmembrane topology. This was conducted with MD simulations that examined binding and conformational changes of two p7 monomers in aqueous solution and at the interface of a complex ER membrane model.

**Fig 7:**
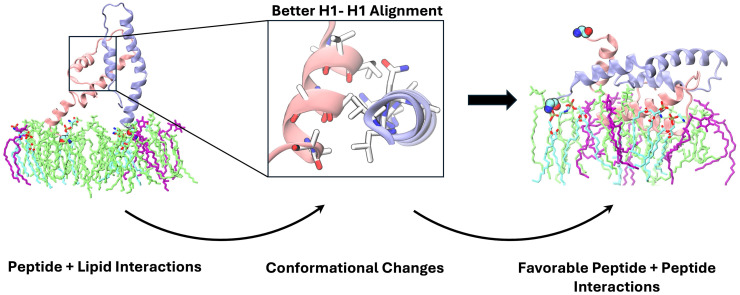
Suggested mechanism of p7 dimerization at the ER membrane surface, based on results from this study. DOPC and POPI lipids shown in light green and purple, helix 1 (H1) residues shown in white, oxygen atoms in red and nitrogen in blue. Monomers 1 and 2 differentiated with pink and ice-blue cartoon representation. Water and ions omitted for clarity during rendering.

Results establish that the presence of lipids prompts better protein alignment at the membrane interface, through the mechanism outlined in Fig 7. During first contact with the membrane, PC and PI lipids enable strong attachment of the monomers through hydrophobic and hydrogen bonding interactions with specific residues, favoring 1-to-1 helix interactions of H1, H2 and H3 from both monomers, as well as H3 and H2 contacts. These contacts stabilize conformations that allow favorable hydrophobic residue interactions, particularly in the case of H1-H1 alignment, as shown by protein helicity, inter-residue contacts, and residue side chain orientation detailed in this study. Taken together with our previous work [21], this study emphasizes the non-trivial role that specific membrane lipid species play in monomer membrane adsorption and dimerization.

This classical MD simulation study also highlights the energy barriers that exist in the process of dimerization. Future work should employ enhanced sampling techniques to provide an in-depth understanding of dynamics and interactions that orchestrate the assembly mechanism of homo-oligomeric channels composed of helical proteins. Such studies will help to elucidate the role of lipids in protein insertion and oligomerization for various viral proteins, as well as those involved in signaling pathways relevant to healthy and other diseased conditions that remain elusive.

## Supporting information

S1 Text Description of analysis. (DOCX)

S1 Table Description of systems studied. The composition of the bilayer in the *Surface* model was set as 55% DOPC, 21% DPPE, 11% POPI, 9% cholesterol and 4% DOPS. Each membrane contained 1200 total lipids (600 per leaflet).
(XLSX)

S2 Table List of protein residues and selected atoms to define the tilt angle vectors. Nomenclature corresponds to the C36m FF [35,40].
(XLSX)

S1 Fig Initial positioning of monomers in replicas 1, 2, 3 and 4 of *Surface* model. Lipid phosphorus atoms shown in green to illustrate positioning with respect to membrane.
(TIFF)

S2 Fig Deviations of the *Bound* model protein dimer conformation from the reference crystal structure of the channel (PDBID: 2M6X). 2D RMSD for **A)** rep1, **B)** rep2, and **C)** rep3. Color bar indicates trajectory time points from 1 to 400 ns. **D)** *Bound* Rep2 monomers aligned to the initial coordinates in the channel conformation; pep1 overlayed onto corresponding monomer in the crystal structure (in grey) is shown in pink, and pep2 in ice-blue.
(TIFF)

S3 Fig Root mean square deviation measurements of monomers. **A)** RMSD of monomer 1 (p1) and **B)** monomer 2 (p2) calculated using the first frame of each protein as the reference in each case. Results for *Sep* model on the left, and *Surface* on the right. **C)** Average RMSD of *Sep* and *Surface* monomers using the *Bound* dimer structure as the reference. **D)** Reference structures used to calculate RMSD. Error bars represent standard error across replicas, and “*” indicates significant difference in means (p < 0.05).
(TIFF)

S4 Fig RMSF per residue in individual p7 monomers for each *Sep* and *Surface* model replica. Most replicas equilibrated after 200 ns (*Sep*) and 500 ns (*Surface*); except Sep R2, Surface R3 and R4. Most systems converged within the last half of the trajectory (200–400 ns in *Sep*, 500–1000 ns in *Surface,* respectively). Time slices are 50 ns each.
(TIFF)

S5 Fig Distance of each amino acid from the average position of phosphorus atoms in the contacting membrane leaflet in the *Surface* systems. The average position of phosphorus atoms is indicated with the red line at 0. Error bars represent standard error across replicas.
(TIFF)

S6 Fig Contact maps of residues during the last half of trajectory (200 ns in *Sep* and 500 ns in *Surface* model), based on a cutoff of 14 Å. Green rectangles indicate region populated in all *Bound* model replicas. Top to bottom panels correspond to either replicas 1–3 (*Bound* and *Sep* models) or 1–4 (*Surface* model).
(TIFF)

S7 Fig Tilt angle conformational landscapes of residues involved in 1-to-1 helix contacts during the last 200 ns of trajectory in the *Sep* model. Results for helices 1, 2 and 3 shown in the left, middle and right columns, and replicas 1, 2 and 3 shown in the top, middle and last rows. Yellow rectangles indicate populated regions conserved across all *Bound* replicas.
(TIFF)

S8 Fig Tilt angle conformational landscapes of residues involved in 1-to-1 helix contacts during the last 500 ns of trajectory in the *Surface* model. Results for helices 1, 2 and 3 shown in the left, middle and right columns, and replicas 1, 2, 3 and 4 shown in the corresponding rows. Yellow rectangles indicate populated regions conserved across all *Bound* replicas.
(TIFF)

S9 Fig Surface replica 4 structural evaluation. Time series of **A)** R_g_ and **B)** SASA of *Surface* replica 4. **C)** Final conformation, and **D)** 27ns snapshot of the conformation of the dimer on the contacting membrane leaflet in this replica; phosphorus atoms are shown in green for reference. Proteins differentiated with pink (p1) and ice-blue (p2), with the N-terminus end indicated with van der Waal representation. In panel D, the residues within 8 Å of the phosphorus atoms are also shown, with nonpolar residues in white, polar in green, cationic in blue.
(TIFF)

S10 Fig Coulomb (electrostatic), Lennard-Jones (hydrophobic), and total interaction energy of interacting monomer helices in each model. Error bars represent standard error across replicas, and “**” indicates significant difference in means (p < 0.01). Only non-zero estimates of p1 and p2 helix interaction energies are shown.
(TIFF)

S11 Fig Snapshot showing cholesterol contacts with the N-terminus of p2 and H2 and H3 of p1 in the *Surface* model. Lipid phosphorus atoms are shown in green as reference, the rest of the lipid structure, water, and ions are hidden for clarity. Proteins differentiated in pink and ice-blue, with the N-terminus end indicated with van der Waal representation. Non-polar and polar protein residues in white and green respectively.
(TIFF)

S12 Fig Frequency of hydrogen bonds between individual residues and lipid species during the last half of trajectory (500 ns) in the *Surface* model. Results shown are for representative *Surface* replicas that form the most accurate dimer contact configuration.
(TIFF)

S13 Fig Protein-lipid dynamic cross correlation maps for *Surface* replicas 2 and 4 during the entire trajectory. Negative and positive correlations represented in blue and red respectively.
(TIFF)
