# Supplementary material for: Lipid-driven alignment and binding of p7 dimers in early oligomer assembly
Source: PLoS Comput Biol. 2025 Nov 25;21(11):e1013736. doi: 10.1371/journal.pcbi.1013736 (PMC12671758; doi:10.1371/journal.pcbi.1013736)
Supplement: S1 Text — (DOCX) [file pcbi.1013736.s001.docx]

**Description of analysis**

*Solvent accessible surface area (SASA)*

SASA quantifies protein packing by taking a measure of the exposed surface area to surrounding solvent. Fully folded proteins with lower SASA values are generally considered more thermodynamically stable (1), making it a useful measure to draw conclusions about protein structure and equilibrium. It can be estimated with the Double Cubic Lattice Method (DCLM) algorithm from Eisenhaber et al, which uses 3 dimensional grids laid over the molecule to group spatially close atoms and indicate unobstructed atoms according to equation 1 (2, 3).

|  | $SASA=4\pi\sum_{i} {r_{i}}^{2}\frac{m_{acc}(i)}{m}$ | (1) |
| --- | --- | --- |

where $m$ is the total number of dots that cover a unit sphere, $m_{acc}(i)$ is the number of dots on atom i not occluded by neighboring atoms, and $r_{i}$ is the atomic radius. The summation is taken over all the atoms in the molecule.

*Radius of gyration (R_g_)*

R_g_ can be used to gain further insight into the compactness of proteins; for example, a smaller R_g_ indicates a more compact molecule (4). In simulations, it aids in determining the thermal stability of the system.  *R_g_* is defined as the square root of the mass-average of the radii components orthogonal to each axis. Over a time series, the evolution of this property can be computed according to equation 2:

|  | $R_{g}= \sqrt{\frac{\sum_{i}m_{i}{r^{2}}_{i}}{\sum_{i}m_{i}}}$ | (2) |
| --- | --- | --- |

where $m_{i}$ is the mass of atom i, and $r_{i}$ is the position of atom i.

*Root mean square deviation and fluctuation (RMSD and RMSF)*

RMSD quantifies shifts in internal coordinates of a structure by first aligning the atoms in each frame of the trajectory with a least-square fit using a user-specified reference frame and computing the distance between the two. RMSF quantifies the average over time of the RMSD to examine individual dynamics of residues or user-selected atoms; here it is presented for each protein residue. Both RMSD and RMSF can be used to determine equilibrium during a simulation trajectory and inform of sampling of specific conformational changes (5). The RMSD is calculated according to equation 3:

|  | $RMSD(t_{1},t_{2})= \sqrt{\frac{1}{M}\sum_{i=1}^{N} m_{i}\left\Vert r_{i}\left( t_{1} \right)-r_{i}\left( t_{2} \right) \right\Vert^{2}}$ | (3) |
| --- | --- | --- |

where $M=\sum_{i=1}^{N} m_{i}$ , $m_{i}$is the mass of atom i and $r_{i}\left( t \right)$ is the position of atom $i$ at time $t$. The reference structure is found at time $t_{2}$; If the initial structure is used as a reference, then $t_{2}$ = 0.

The RMSF is calculated as the time averaged value of the RMSD for each of the protein residues or user-selected atoms.

*Helicity*

Helicity is a measure of the percentage of the amino acids in a user-defined selection that meet the biochemical criteria to be considered part of a helical structure. In this work, the criteria is based on the STRIDE algorithm, which uses a weighted contribution of hydrogen bonding patterns, energy and backbone torsion angle probabilities to define thresholds that fulfill the minimal conditions of secondary structure. Equations outlining the criteria are found in the methods section of (6).

*Residue-lipid hydrogen bonds*

Residue-lipid hydrogen bond analysis measures the strength of interactions between each residue and each type of phospholipid in the membrane by quantifying the frequency of hydrogen bonds formed over a chosen time slice. Hydrogen bonds are identified using the VMD *hbonds* plugin, where a bond is counted when the distance between the bond donor and the bond acceptor is less than 3.2 Å, and the angle between the donor to the hydrogen to the acceptor (angle D--H-A) is less than 30^o^ (7).

*Tilt angle conformational landscape*

The conformational landscapes of protein residues give an understanding of how residue conformation changed in hand with sustained interactions between monomers. The algorithm to calculate this is as follows:

1. When a residue of the first monomer is within 14 Å of another in the second monomer, a contact is defined.
2. Strongly contacting residues are filtered based on if the frequency of contact in the time slice considered is greater than 0.9.
3. Reference vectors for each protein helix are defined using residues of low flexibility that hardly change in helical conformation.
4. Tilt angle vectors are defined as the vector connecting each residue’s alpha carbon and its terminal tail atom outlined in Table S1.
5. Using the vectors, the tilt angle of the residues that meet the criteria are plotted as coordinates on a 2D plane.


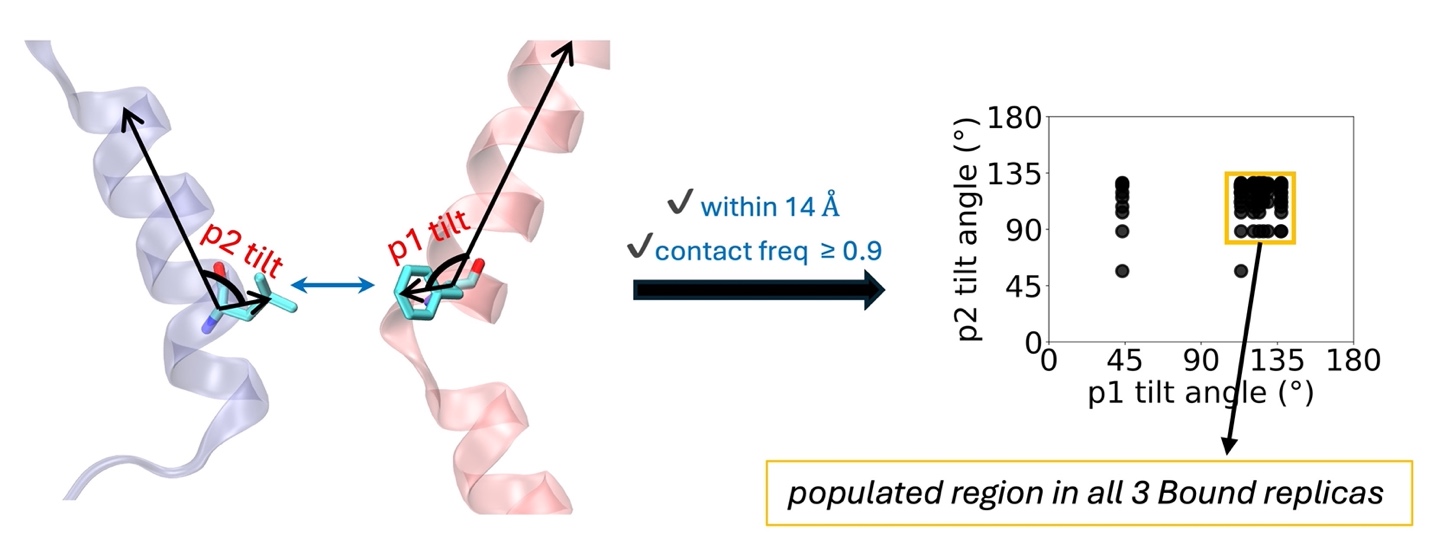


The figure above summarizes how the vectors and criteria were defined and provides guidance on how to interpret the measured tilt angles of a sample 2D plot.

*Dynamic cross correlation (DCC) analysis*

DCC is used to identify parts of a protein that experience correlated movement. This value can be positive, which indicates movement in the same direction, or negative, which indicates movement in opposite directions. The algorithm used in this work is adapted from Tekpinar et al (8), measuring the displacement of the protein alpha carbons relative to that of lipid headgroup phosphorus atoms within 20 Å of each other. The time average of the dot product between the positional vectors for each carbon-phosphorus combination was computed according to equation 4:

|  | ${DCC}_{ij}=\left\langle\Delta\boldsymbol{R}_{\boldsymbol{i}}\cdot\Delta\boldsymbol{R}_{\boldsymbol{j}} \right\rangle$ | (4) |
| --- | --- | --- |

where $\Delta\boldsymbol{R}_{\boldsymbol{x}}=\boldsymbol{R}_{\boldsymbol{x}}- \left\langle\boldsymbol{R}_{\boldsymbol{x}} \right\rangle$is the positional difference vector of atom *i* with respect to its average position after an overall root mean square alignment procedure has been conducted.

*Binding free energy estimates*

Here, we use the Molecular Mechanics Generalized-Born Surface Area (MM-GBSA) method to estimate the energetic cost that must be overcome for protein binding. The binding free energy was calculated according to the following equations:

|  | ${\Delta G}_{binding}=\Delta G_{MM}+\Delta G_{solv}-T\Delta S$ | (5) |
| --- | --- | --- |
|  | ${\Delta G}_{MM}= G_{MM,complex}- G_{MM,receptor} - G_{MM,ligand}$ | (6) |
|  | $G_{MM,i}= {{(E}_{bonded}+E_{elect}+E_{vdW})}_{i}$ | (7) |
|  | ${\Delta G}_{solv}= {\Delta G}_{el}+ {\Delta G}_{surf}$ | (8) |

where ${\Delta G}_{MM}$ represents the difference in sum of the bonded ($E_{bonded}$) and nonbonded (electrostatic, $E_{elect}$ and van der Waals, $E_{vdW}$) interaction energy of the complexed molecule and those of the individual components, which are usually termed the receptor and ligand respectively (9). The ${\Delta G}_{solv}$ is the solvation energy, which estimates the polar contribution, ${\Delta G}_{el}$ with the Generalized-Born (10) equation, and the nonpolar contribution, ${\Delta G}_{surf}$ is based on a linear approximation of the solvent accessible surface area of the complex (11, 12).

**References**

1. Lee Y, Lee J-j, Kim S, Lee S-C, Han J, Heu W, et al. Dissecting the Critical Factors for Thermodynamic Stability of Modular Proteins Using Molecular Modeling Approach. PLOS ONE. 2014;9(5):e98243.

2. GROMACS. gmx sasa 2024 [Available from: <https://manual.gromacs.org/2024.2/onlinehelp/gmx-sasa.html>.

3. Eisenhaber F, Lijnzaad P, Argos P, Sander C, Scharf M. The Double Cubic Lattice Method - Efficient Approaches to Numerical-Integration of Surface-Area and Volume and to Dot Surface Contouring of Molecular Assemblies. J Comput Chem. 1995;16(3):273-84.

4. GROMACS. Radius of gyration and distances 2024 [Available from: <https://manual.gromacs.org/current/reference-manual/analysis/radius-of-gyration.html>.

5. GROMACS. Root mean square deviations in structure 2024 [Available from: <https://manual.gromacs.org/current/reference-manual/analysis/rmsd.html>.

6. Frishman D, Argos P. Knowledge-based protein secondary structure assignment. Proteins: Struct Funct Genet. 1995;23(4):566-79.

7. Gumbart J, Luo D. HBonds Plugin, Version 1.2 2011 [Available from: <https://www.ks.uiuc.edu/Research/vmd/plugins/hbonds/>.

8. Tekpinar M, Neron B, Delarue M. Extracting Dynamical Correlations and Identifying Key Residues for Allosteric Communication in Proteins by correlationplus. J Chem Inf Model. 2021;61(10):4832-8.

9. Genheden S, Ryde U. The MM/PBSA and MM/GBSA methods to estimate ligand-binding affinities. Expert Opin Drug Discov. 2015;10(5):449-61.

10. Onufriev A, Bashford D, Case DA. Exploring protein native states and large-scale conformational changes with a modified generalized born model. Proteins: Struct, Funct, Bioinf. 2004;55(2):383-94.

11. Valdés-Tresanco MS, Valdés-Tresanco ME, Valiente PA, Moreno E. gmx_MMPBSA: A New Tool to Perform End-State Free Energy Calculations with GROMACS. J Chem Theory Comput. 2021;17(10):6281-91.

12. Sitkoff D, Sharp KA, Honig B. Accurate Calculation of Hydration Free Energies Using Macroscopic Solvent Models. J Phys Chem. 1994;98(7):1978-88.
