# Supplementary figures and images for: Lipid-driven alignment and binding of p7 dimers in early oligomer assembly
Source: PLoS Comput Biol. 2025 Nov 25;21(11):e1013736. doi: 10.1371/journal.pcbi.1013736 (PMC12671758; doi:10.1371/journal.pcbi.1013736)

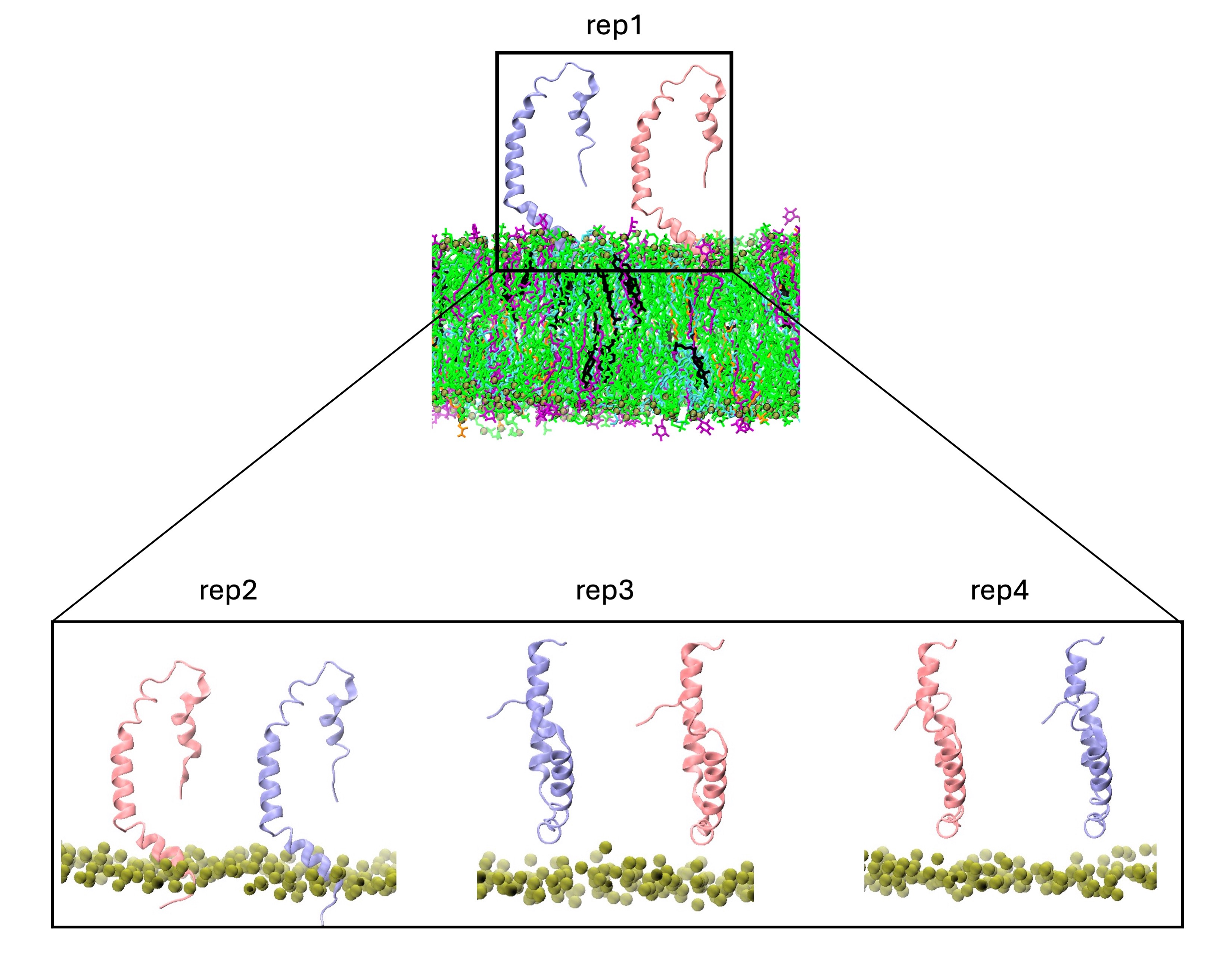

Supplement: S1 Fig — Lipid phosphorus atoms shown in green to illustrate positioning with respect to membrane. (TIFF) [file pcbi.1013736.s004.tiff]

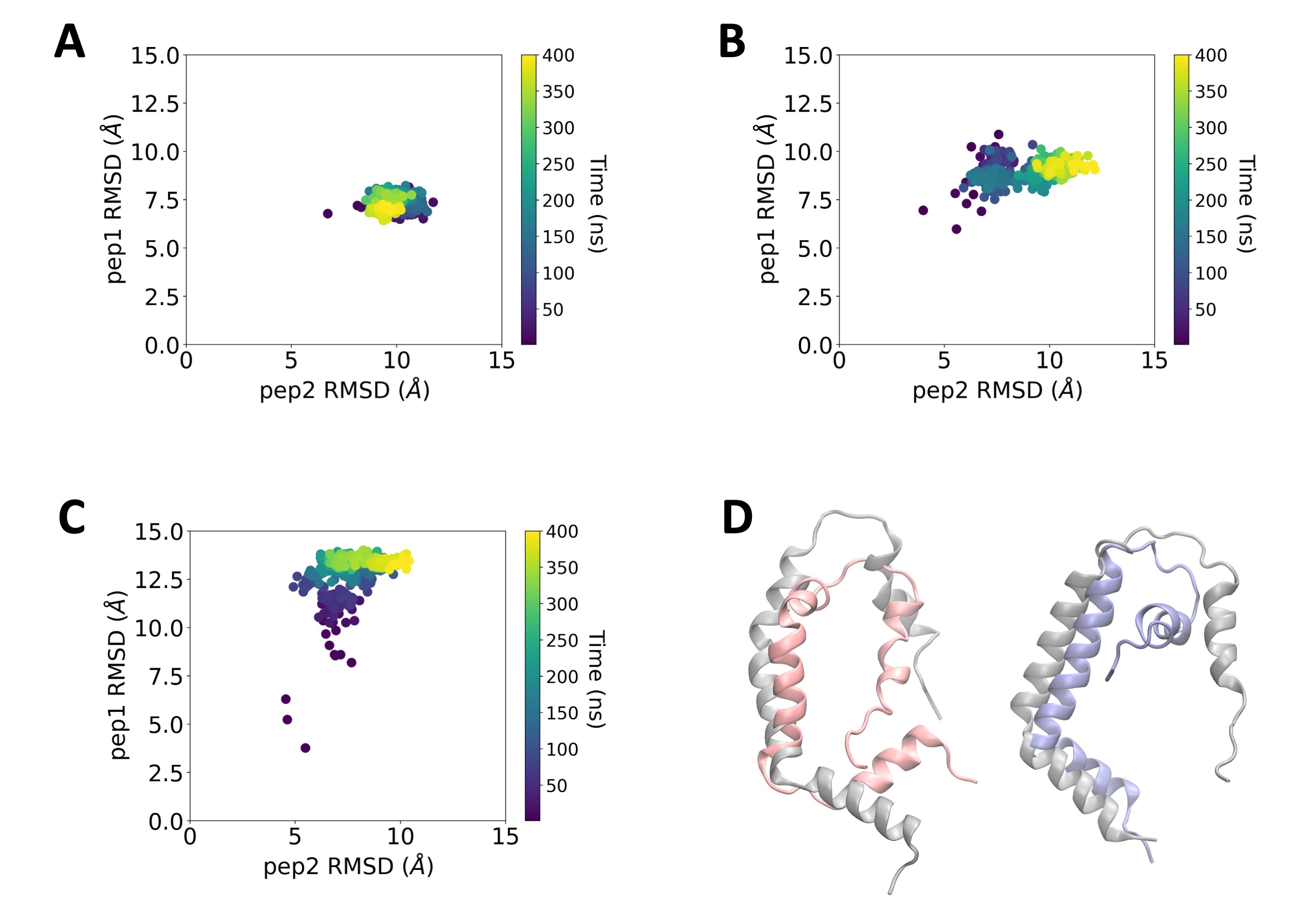

Supplement: S2 Fig — 2D RMSD for A) rep1, B) rep2, and C) rep3. Color bar indicates trajectory time points from 1 to 400 ns. D) Bound Rep2 monomers aligned to the initial coordinates in the channel conformation; pep1 overlayed onto corresponding monomer in the crystal structure (in grey) is shown in pink, and pep2 in ice-blue. (TIFF) [file pcbi.1013736.s005.tiff]

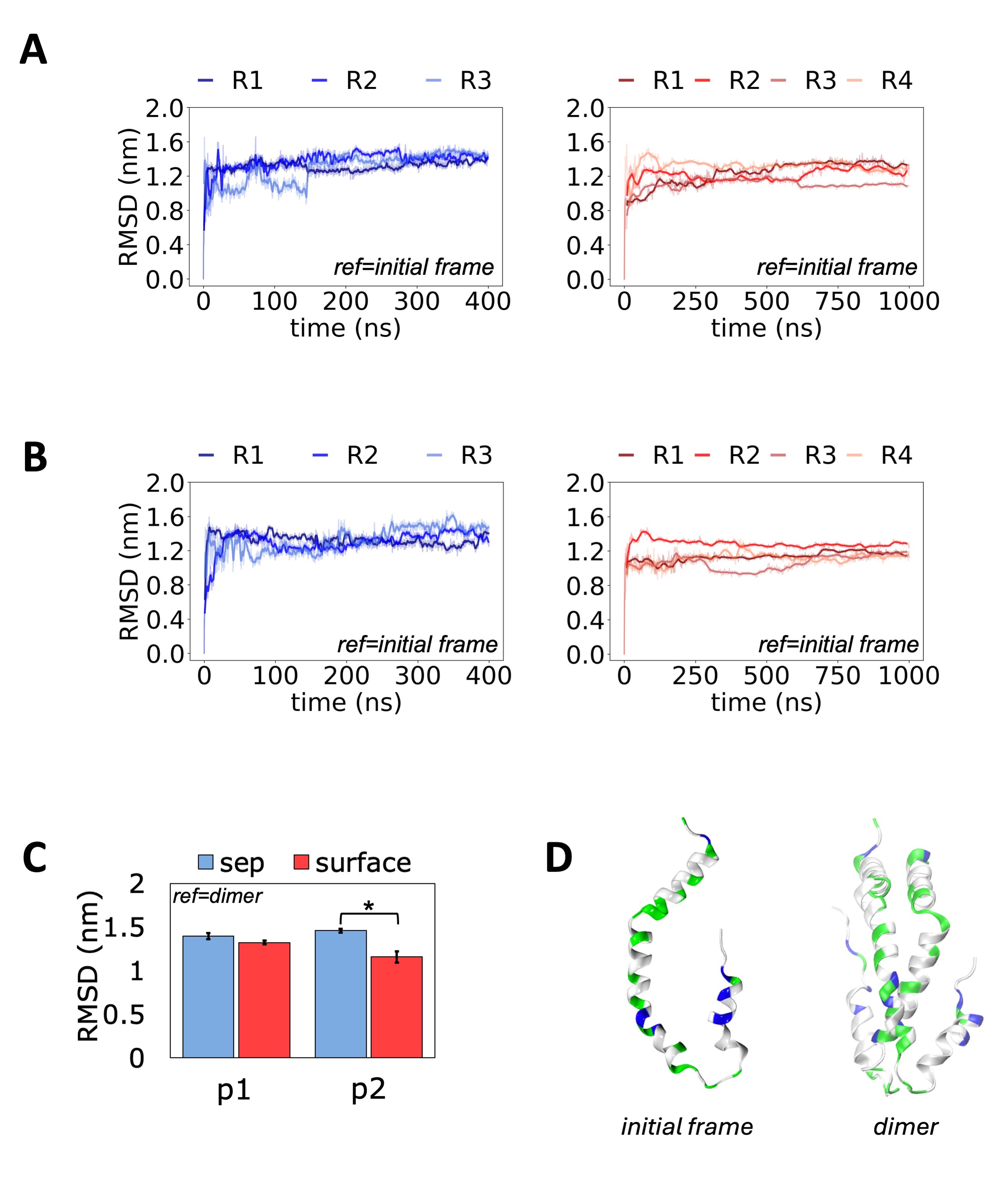

Supplement: S3 Fig — A) RMSD of monomer 1 (p1) and B) monomer 2 (p2) calculated using the first frame of each protein as the reference in each case. Results for Sep model on the left, and Surface on the right. C) Average RMSD of Sep and Surface monomers using the Bound dimer structure as the reference. D) Reference structures used to calculate RMSD. Error bars represent standard error across replicas, and “*” indicates significant difference in means (p < 0.05). (TIFF) [file pcbi.1013736.s006.tiff]

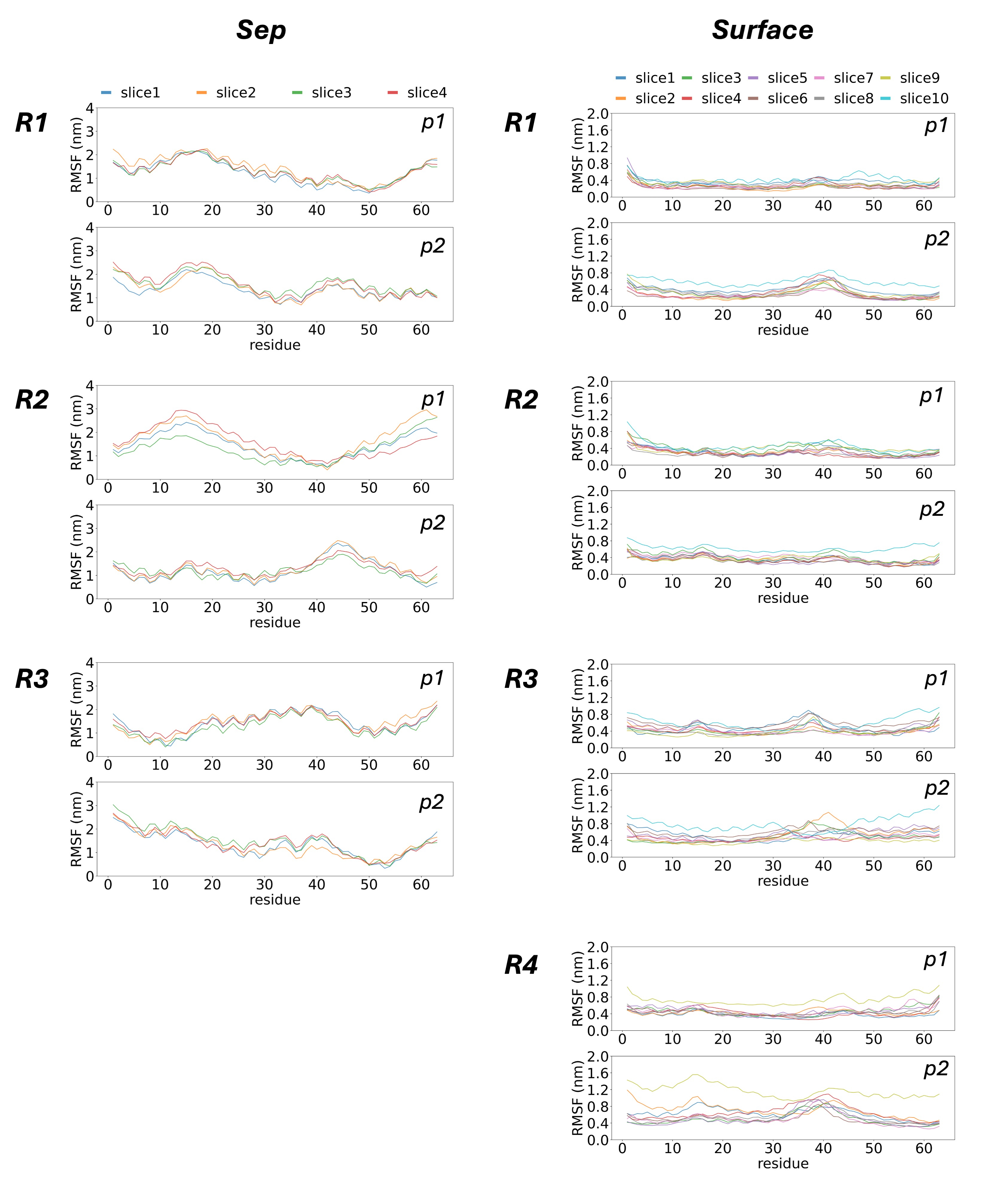

Supplement: S4 Fig — Most replicas equilibrated after 200 ns (Sep) and 500 ns (Surface); except Sep R2, Surface R3 and R4. Most systems converged within the last half of the trajectory (200–400 ns in Sep, 500–1000 ns in Surface, respectively). Time slices are 50 ns each. (TIFF) [file pcbi.1013736.s007.tiff]

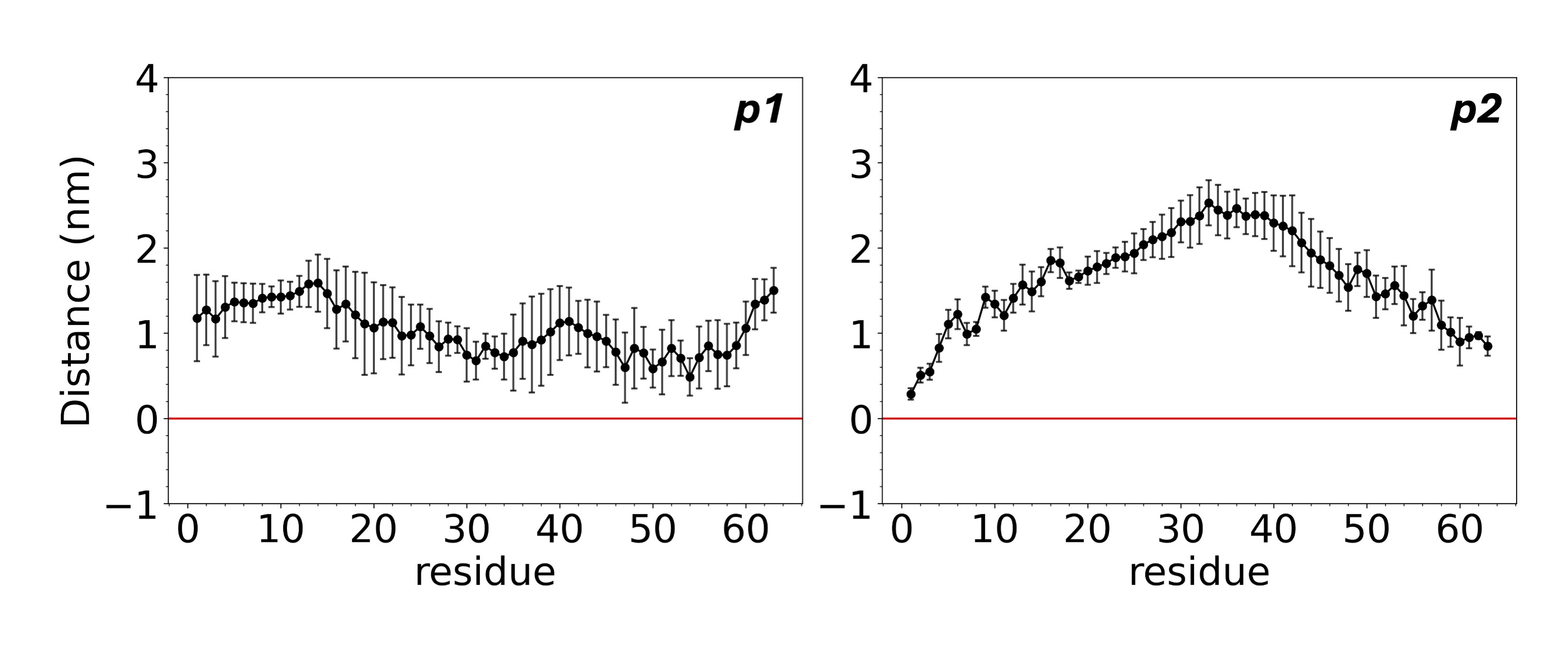

Supplement: S5 Fig — The average position of phosphorus atoms is indicated with the red line at 0. Error bars represent standard error across replicas. (TIFF) [file pcbi.1013736.s008.tiff]

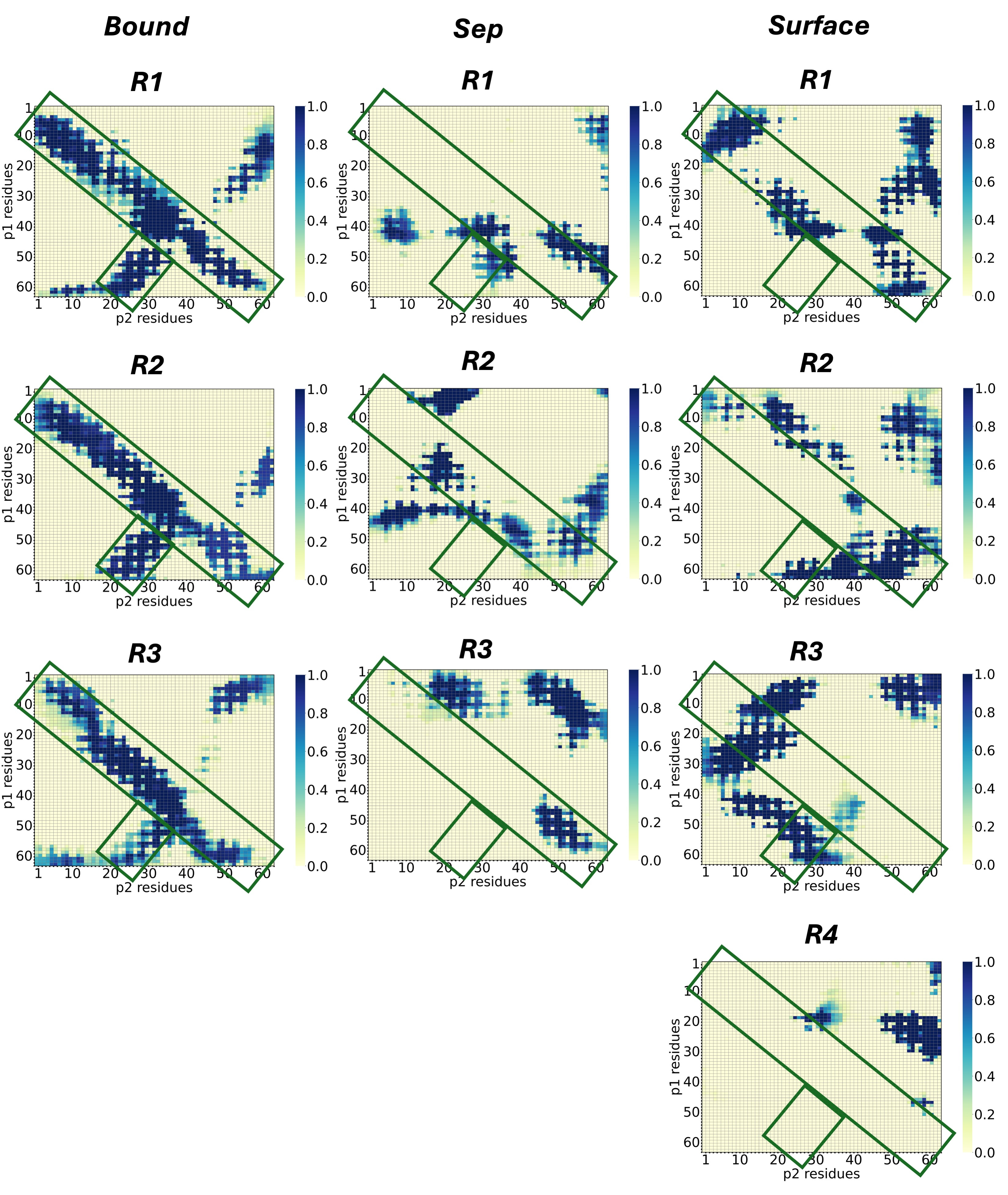

Supplement: S6 Fig — Green rectangles indicate region populated in all Bound model replicas. Top to bottom panels correspond to either replicas 1–3 (Bound and Sep models) or 1–4 (Surface model). (TIFF) [file pcbi.1013736.s009.tiff]

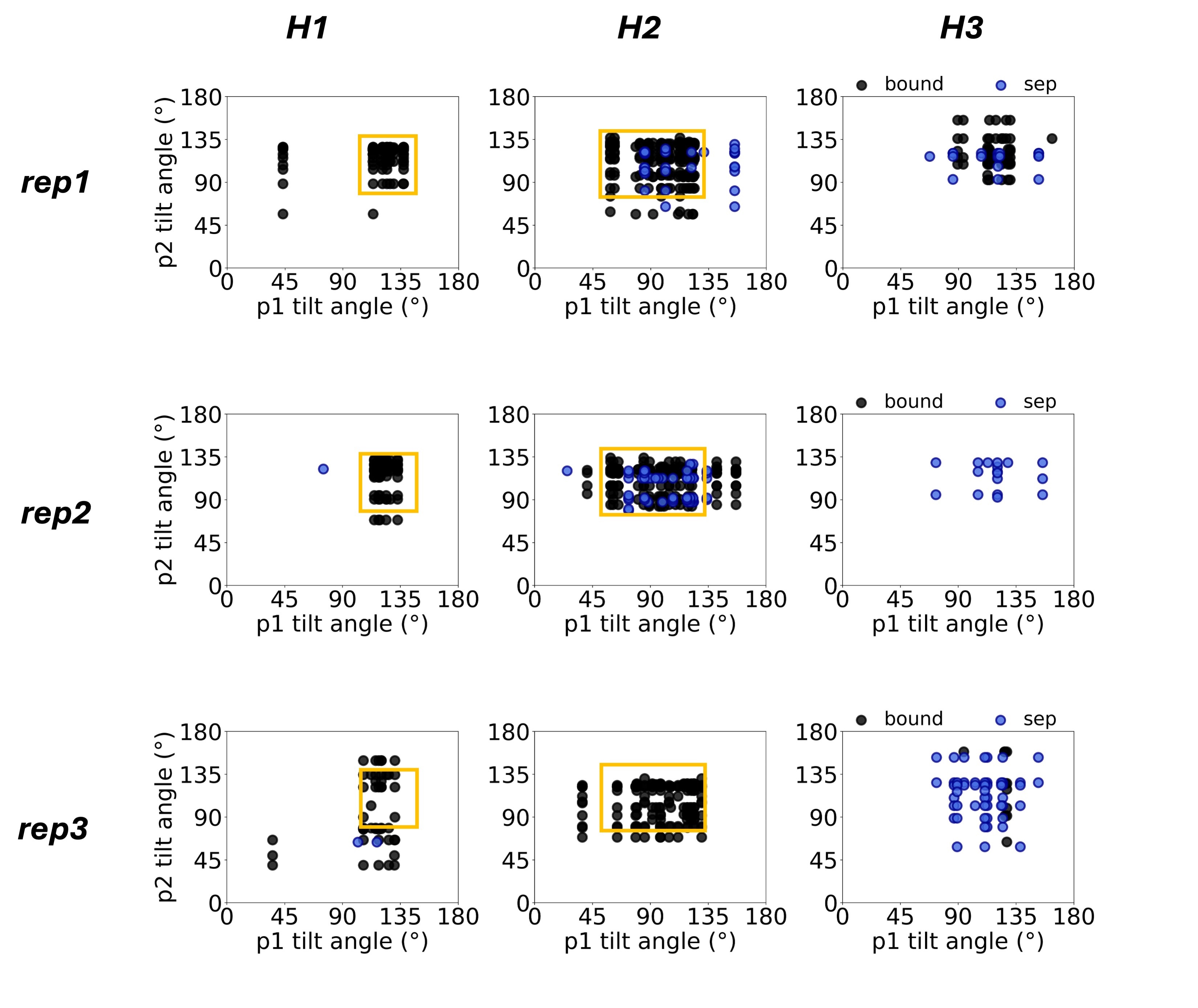

Supplement: S7 Fig — Results for helices 1, 2 and 3 shown in the left, middle and right columns, and replicas 1, 2 and 3 shown in the top, middle and last rows. Yellow rectangles indicate populated regions conserved across all Bound replicas. (TIFF) [file pcbi.1013736.s010.tiff]

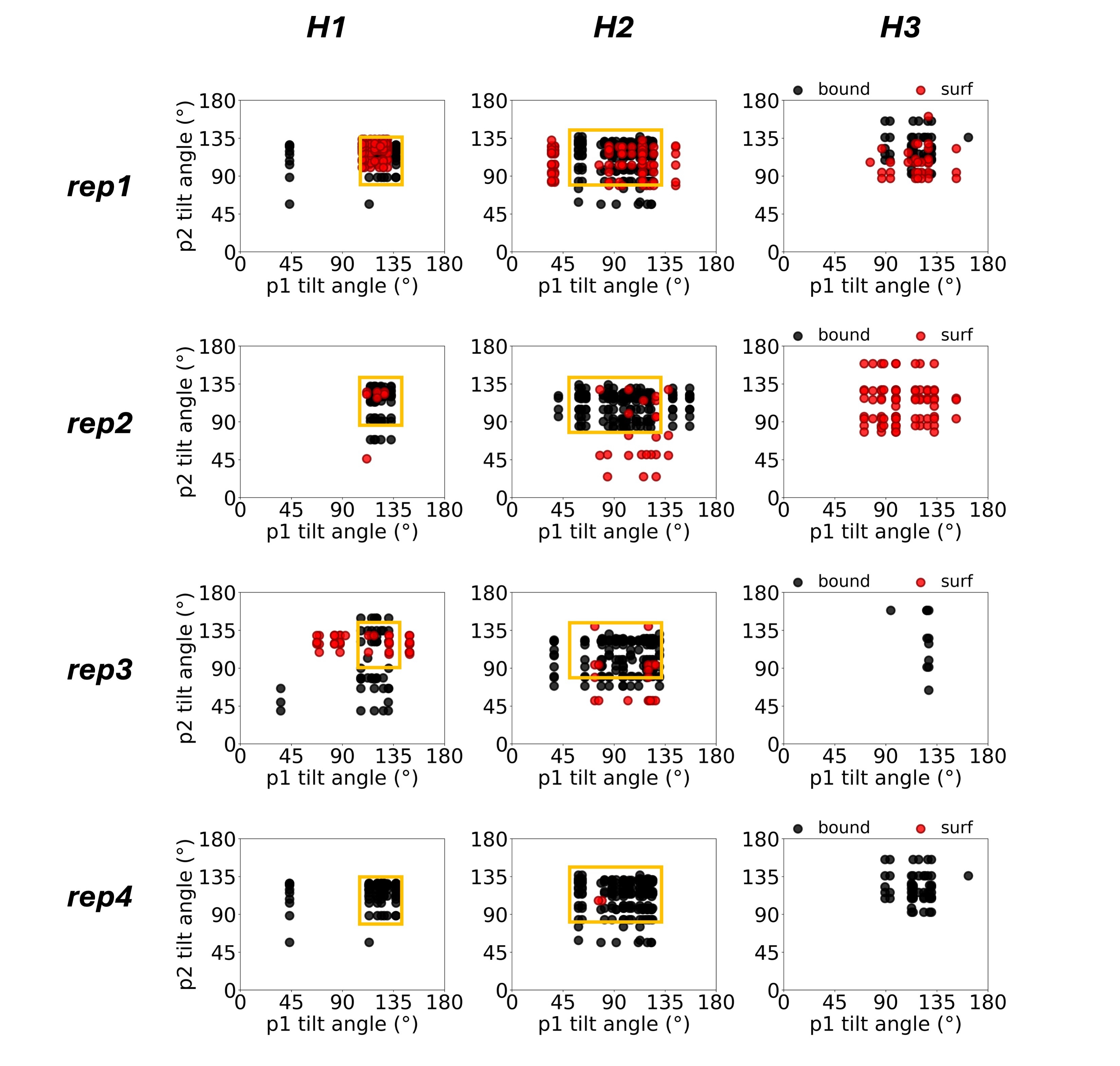

Supplement: S8 Fig — Results for helices 1, 2 and 3 shown in the left, middle and right columns, and replicas 1, 2, 3 and 4 shown in the corresponding rows. Yellow rectangles indicate populated regions conserved across all Bound replicas. (TIFF) [file pcbi.1013736.s011.tiff]

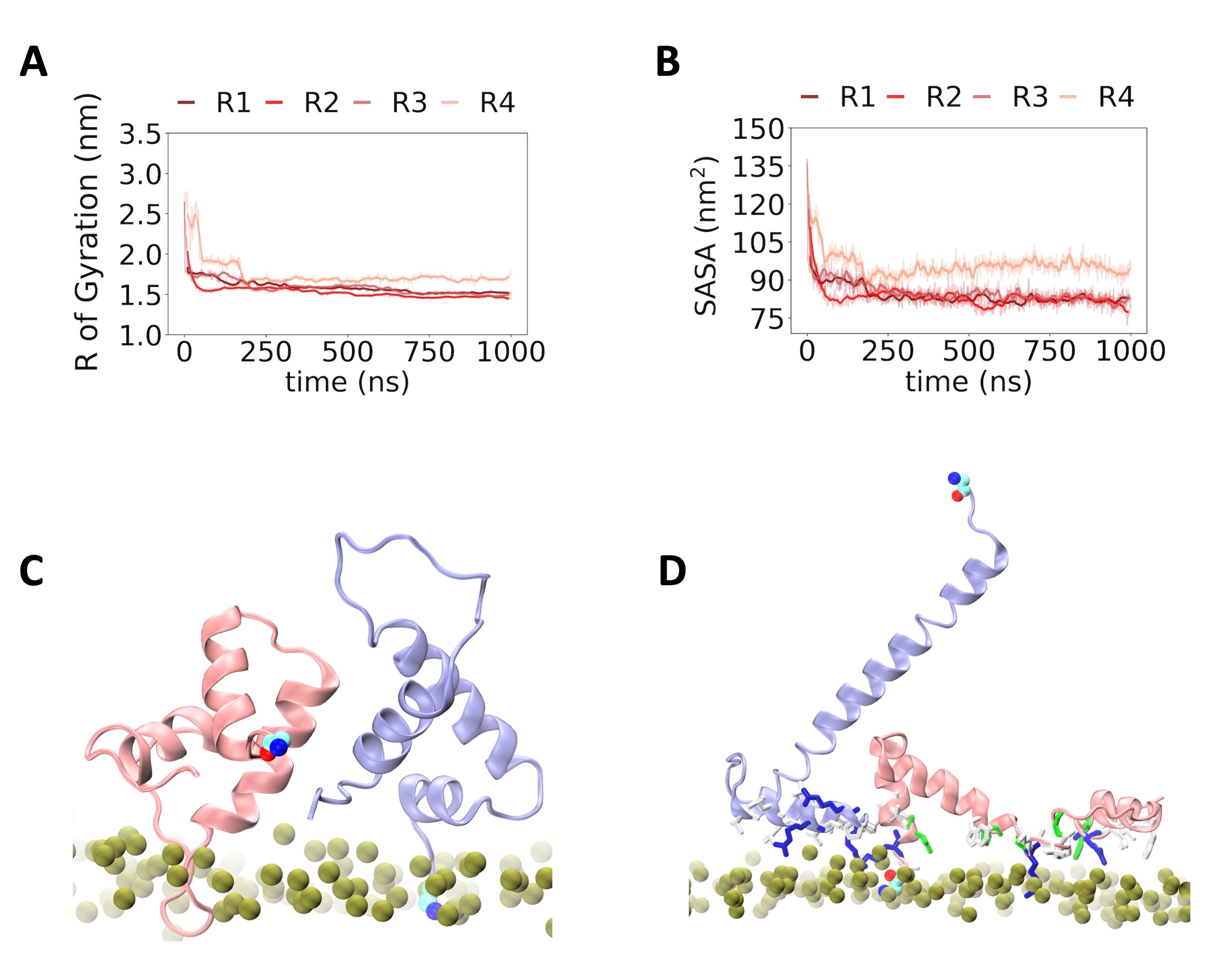

Supplement: S9 Fig — Time series of A) Rg and B) SASA of Surface replica 4. C) Final conformation, and D) 27ns snapshot of the conformation of the dimer on the contacting membrane leaflet in this replica; phosphorus atoms are shown in green for reference. Proteins differentiated with pink (p1) and ice-blue (p2), with the N-terminus end indicated with van der Waal representation. In panel D, the residues within 8 Å of the phosphorus atoms are also shown, with nonpolar residues in white, polar in green, cationic in blue. (TIFF) [file pcbi.1013736.s012.tiff]

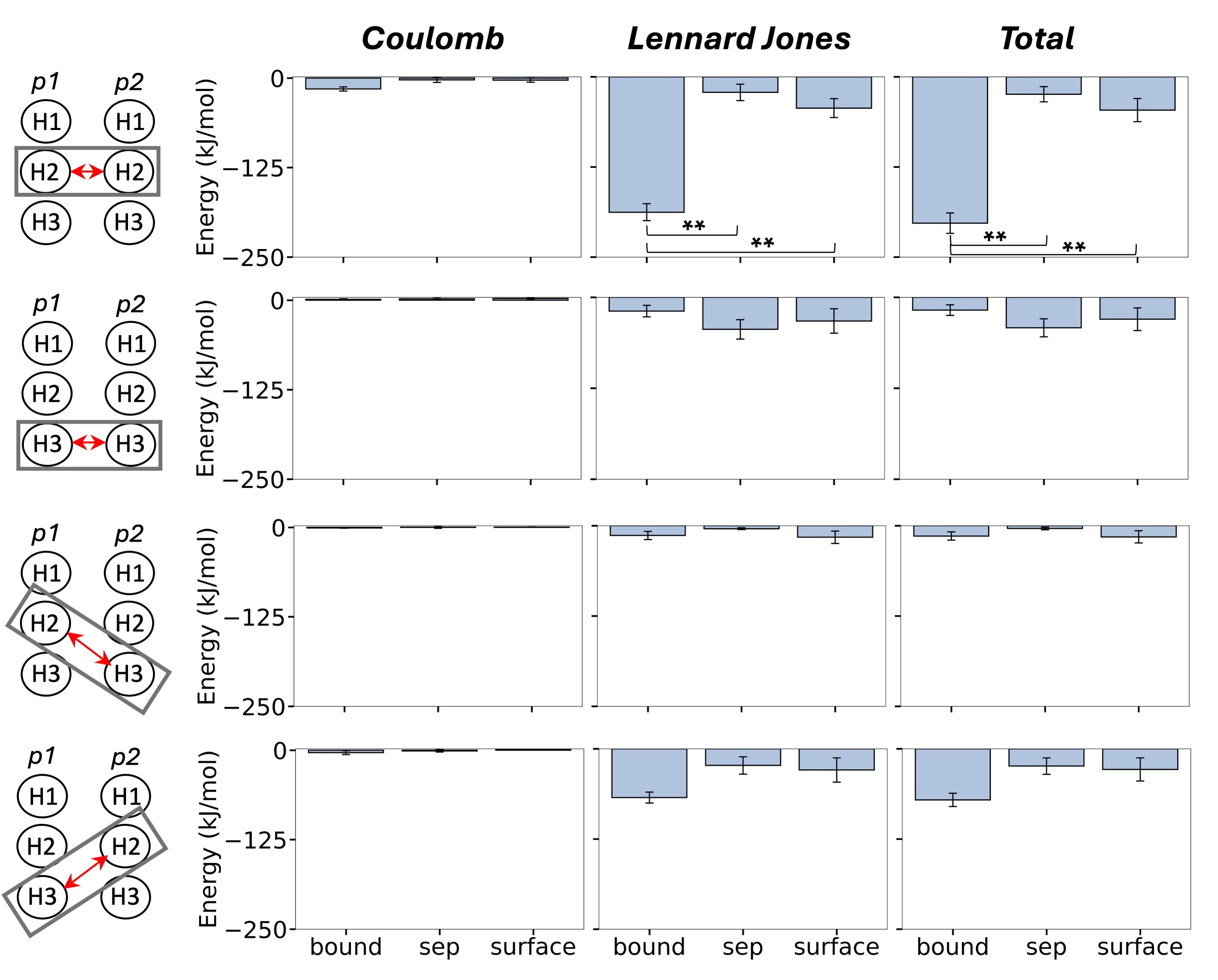

Supplement: S10 Fig — Error bars represent standard error across replicas, and “**” indicates significant difference in means (p < 0.01). Only non-zero estimates of p1 and p2 helix interaction energies are shown. (TIFF) [file pcbi.1013736.s013.tiff]

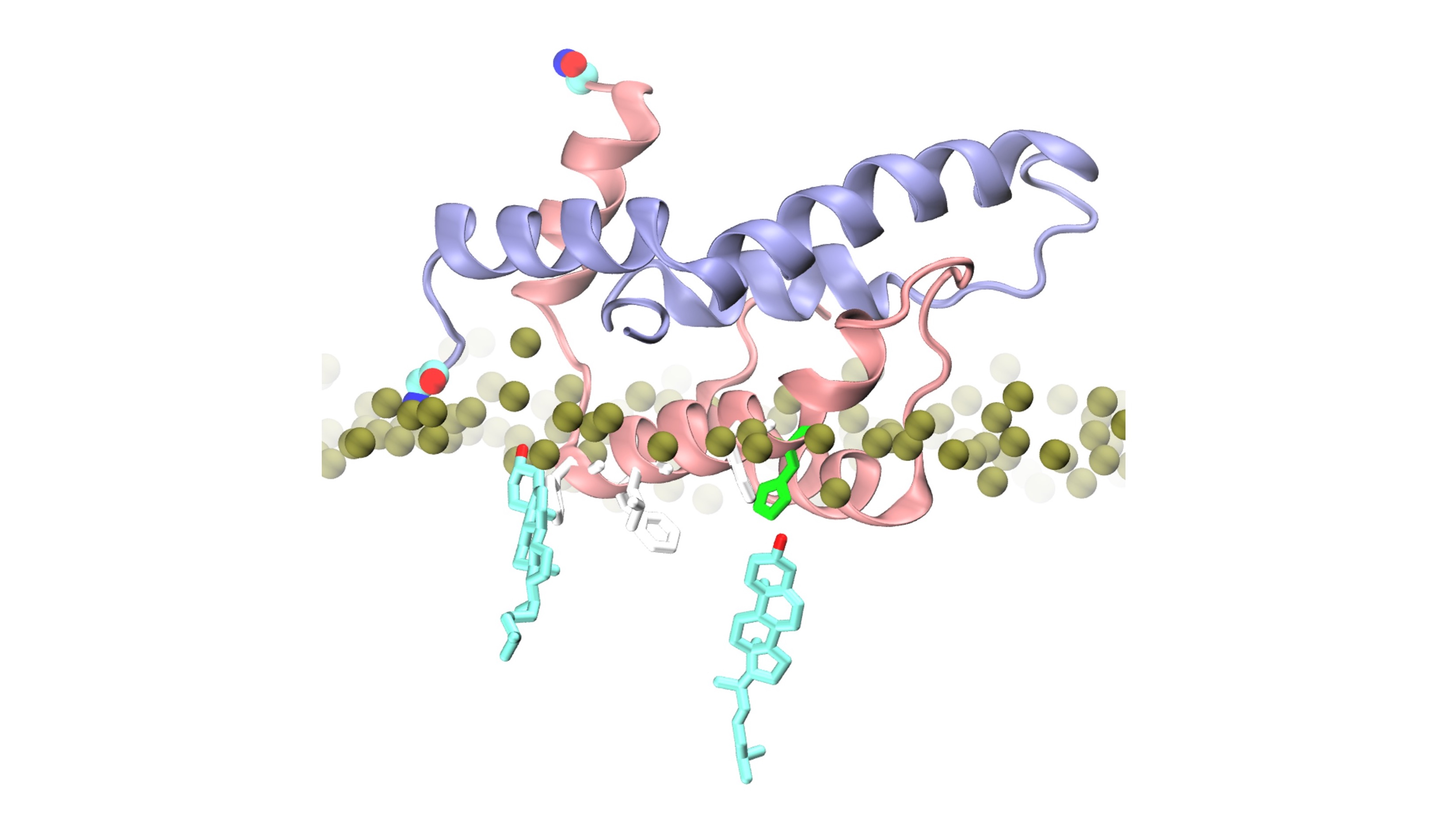

Supplement: S11 Fig — Lipid phosphorus atoms are shown in green as reference, the rest of the lipid structure, water, and ions are hidden for clarity. Proteins differentiated in pink and ice-blue, with the N-terminus end indicated with van der Waal representation. Non-polar and polar protein residues in white and green respectively. (TIFF) [file pcbi.1013736.s014.tiff]

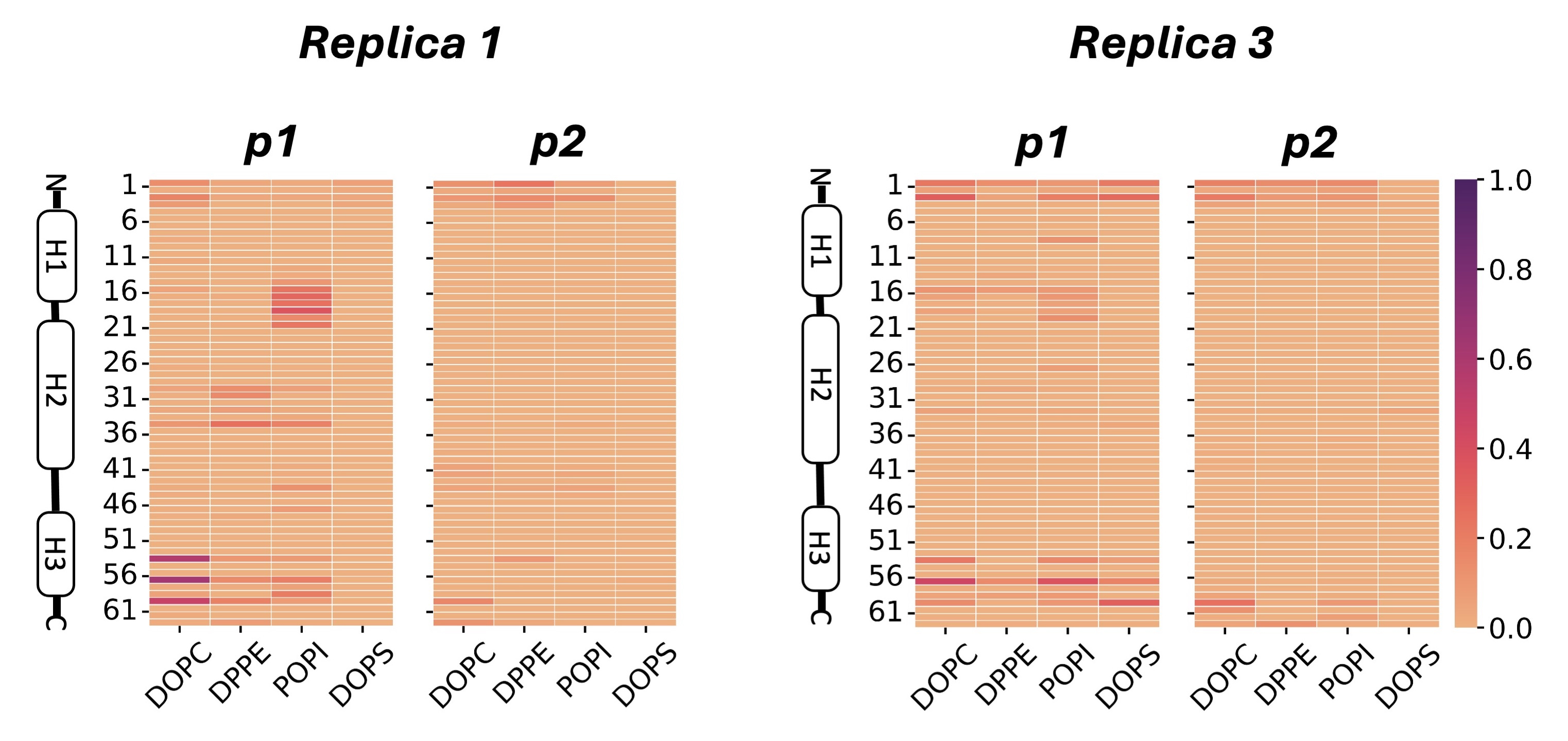

Supplement: S12 Fig — Results shown are for representative Surface replicas that form the most accurate dimer contact configuration. (TIFF) [file pcbi.1013736.s015.tiff]

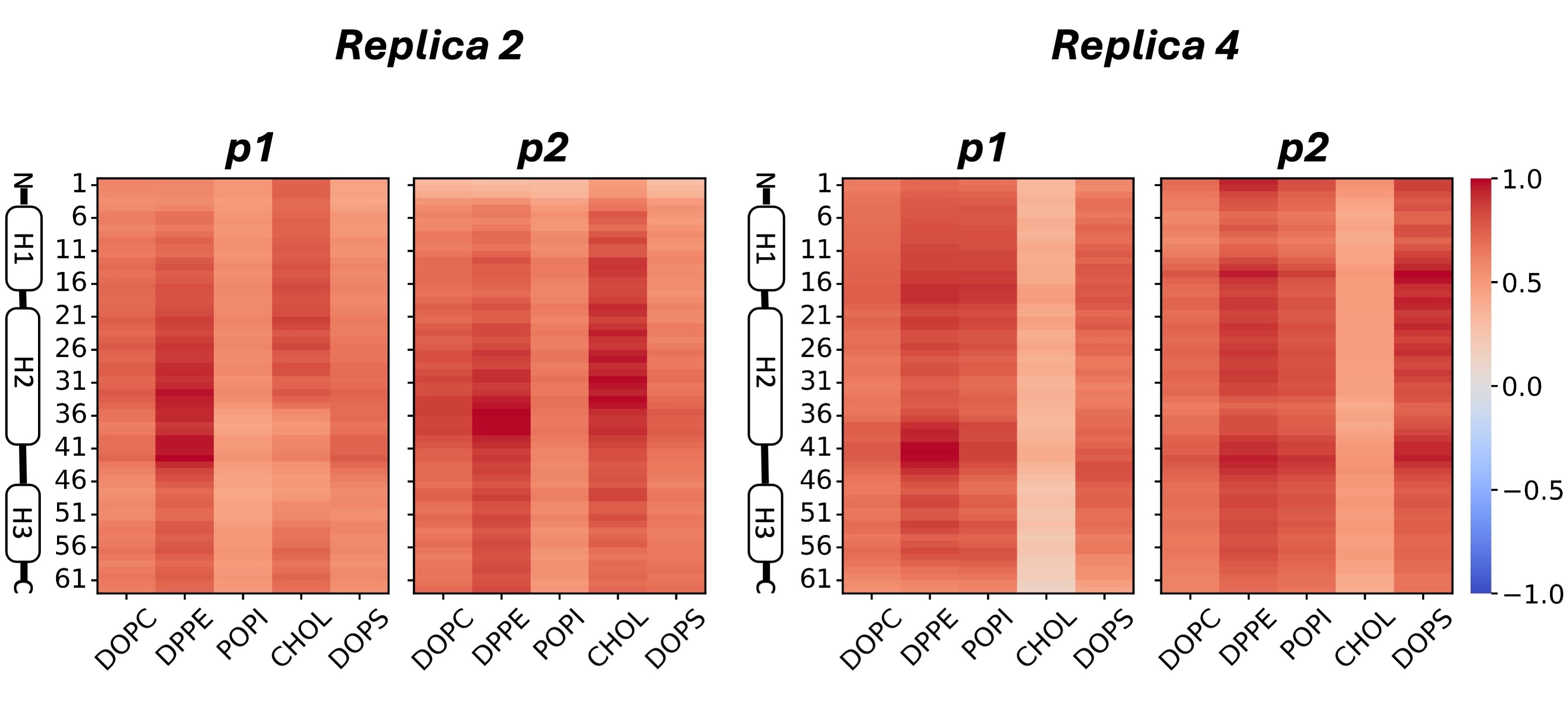

Supplement: S13 Fig — Negative and positive correlations represented in blue and red respectively. (TIFF) [file pcbi.1013736.s016.tiff]
